# Titanium biomaterials with complex surfaces induced aberrant peripheral circadian rhythms in bone marrow mesenchymal stromal cells

**DOI:** 10.1371/journal.pone.0183359

**Published:** 2017-08-17

**Authors:** Nathaniel Hassan, Kirstin McCarville, Kenzo Morinaga, Cristiane M. Mengatto, Peter Langfelder, Akishige Hokugo, Yu Tahara, Christopher S. Colwell, Ichiro Nishimura

**Affiliations:** 1 Weintraub Center for Reconstructive Biotechnology, UCLA School of Dentistry, Los Angeles, California, United States of America; 2 Division of Oral Biology & Medicine, UCLA School of Dentistry, Los Angeles, California, United States of America; 3 Division of Advanced Prosthodontics, UCLA School of Dentistry, Los Angeles, California, United States of America; 4 Department of Oral Rehabilitation, Section of Oral Implantology, Fukuoka Dental College, Fukuoka, Japan; 5 Department of Conservative Dentistry, School of Dentistry Federal University of Rio Grande do Sul, Porto Alegre, Rio Grande do Sul, Brazil; 6 Department of Human Genetics, David Geffen School of Medicine at UCLA, Los Angeles, California, United States of America; 7 Division of Plastic Surgery, David Geffen School of Medicine at UCLA, Los Angeles, California, United States of America; 8 Department of Psychiatry & Biobehavioral Science, David Geffen School of Medicine at UCLA, Los Angeles, California, United States of America; University of Texas Southwestern Medical Center, UNITED STATES

## Abstract

Circadian rhythms maintain a high level of homeostasis through internal feed-forward and -backward regulation by core molecules. In this study, we report the highly unusual peripheral circadian rhythm of bone marrow mesenchymal stromal cells (BMSCs) induced by titanium-based biomaterials with complex surface modifications (Ti biomaterial) commonly used for dental and orthopedic implants. When cultured on Ti biomaterials, human BMSCs suppressed circadian *PER1* expression patterns, while *NPAS2* was uniquely upregulated. The Ti biomaterials, which reduced *Per1* expression and upregulated *Npas2*, were further examined with BMSCs harvested from *Per1*::*luc* transgenic rats. Next, we addressed the regulatory relationship between *Per1* and *Npas2* using BMSCs from *Npas2* knockout mice. The *Npas2* knockout mutation did not rescue the Ti biomaterial-induced *Per1* suppression and did not affect *Per2*, *Per3*, *Bmal1* and *Clock* expression, suggesting that the Ti biomaterial-induced *Npas2* overexpression was likely an independent phenomenon. Previously, vitamin D deficiency was reported to interfere with Ti biomaterial osseointegration. The present study demonstrated that vitamin D supplementation significantly increased *Per1*::*luc* expression in BMSCs, though the presence of Ti biomaterials only moderately affected the suppressed *Per1*::*luc* expression. Available *in vivo* microarray data from femurs exposed to Ti biomaterials in vitamin D-deficient rats were evaluated by weighted gene co-expression network analysis. A large co-expression network containing *Npas2*, *Bmal1*, and *Vdr* was observed to form with the Ti biomaterials, which was disintegrated by vitamin D deficiency. Thus, the aberrant BMSC peripheral circadian rhythm may be essential for the integration of Ti biomaterials into bone.

## Introduction

Titanium (Ti)-based biomaterials are increasingly used in clinical applications for endosseous implants in aging patient populations. The replacement of knee, hip and tooth structure and function by Ti implants may improve patient quality of life and longevity. Ti biomaterials exhibit suitable mechanical properties, such as high strength and a low Young’s modulus [1, 2], as well as excellent biocompatibility, low cytotoxicity and minimal immunogenicity [3]. The therapeutic outcome of successful endosseous implants commonly achieved by direct bonding of regenerating bone to the surface of Ti biomaterials is also called osseointegration [4, 5]. Bone marrow mesenchymal stromal cells (BMSCs) are believed to be primarily responsible for achieving osseointegration. The surface topography at the submicron to micron levels has been extensively investigated with respect to the modulatory effects of BMSCs to improve osseointegration [6, 7]. More recently, methods to create nanometer-scale topographies have been introduced, and the combination of micro/nano-scale topography has been shown to promote osseointegration [8–10].

Implant loosening due to osseointegration failures can occur during the early surgical wound healing stage or after the implant has been in function for some duration [11, 12]. Once osseointegration is lost, therapeutic options are limited to surgically remove the implant. The increased financial burden of implant revision surgery has become a challenge in healthcare systems [13]. Recent pre-clinical and clinical reports suggest that adequate serum vitamin D levels are a critical parameter for the therapeutic success of Ti implants [14–19]. A genome-wide microarray analysis of femur bone marrow tissue exposed to a Ti implant in rats with vitamin D deficiency revealed that the circadian rhythm pathway and, in particular, the expression of Neuronal PAS domain-containing protein 2 (*Npas2*) was most significantly modulated during the Ti implant to bone integration period [20].

Circadian rhythms are generated by an internal clock localized in the suprachiasmatic nucleus (SCN), and their disruption has been reported to cause a wide range of physiological, mental and behavioral disorders [21, 22]. The SCN contains a collection of cell-autonomous oscillators that are regulated by intracellular feed-back and -forward loops involving the transcription of period circadian protein homolog (*PER*) and cryptochrome (*CRY*) genes that are activated by brain and muscle ARNT-like 1 (*BMAL1*) and Circadian locomotor output cycles kaput (*CLOCK*) nuclear protein dimers [23, 24]. In addition to the central circadian rhythm, peripheral tissues are also reported to possess an independent circadian regulatory mechanism to allow greater flexibility in adapting to local environments [25].

This study examined the peripheral circadian rhythms of BMSCs in relation to Ti biomaterials and vitamin D supplementation. In this study, we report evidence that Ti biomaterials, particularly those with complex surface topographies, can peripherally influence the BMSC circadian rhythms robustly.

## Materials and methods

### Ethics statement

All of the experimental protocols using animals were reviewed and approved by the UCLA Animal Research Committee (ARC# 1997–136) and followed the PHS Policy for the Humane Care and Use of Laboratory Animals and the UCLA Animal Care and Use Training Manual guidelines. All of the animals had free access to food and water and were maintained in regular housing with a 12-h light/dark cycle at the Division of Laboratory Animal Medicine, UCLA.

### Titanium (Ti) substrates

**Fig 1:**
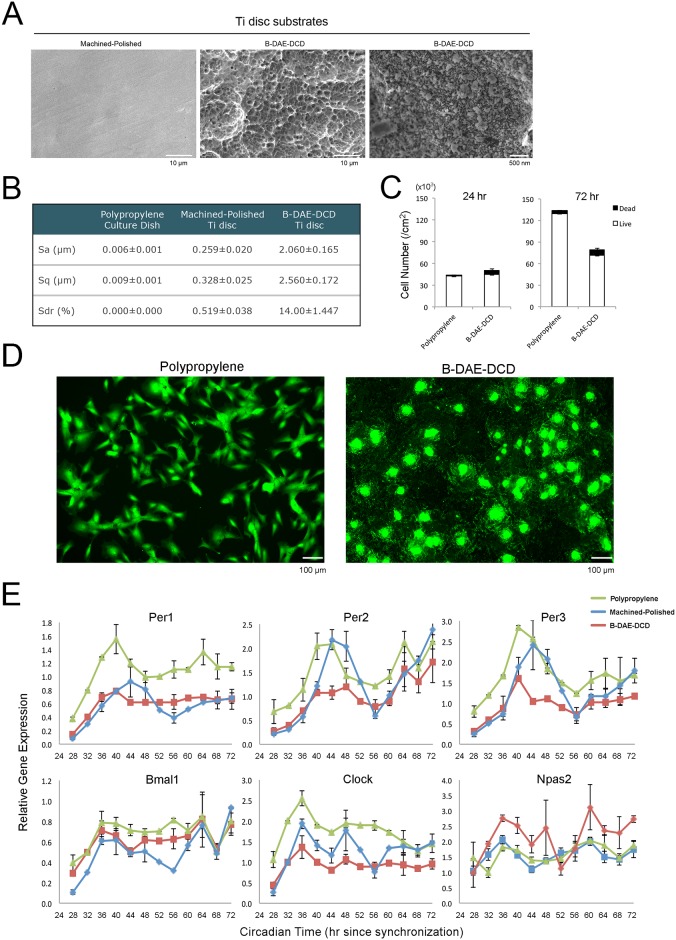
Titanium (Ti)-based biomaterials significantly modulated the expression patterns of circadian rhythm genes from human bone marrow stromal cells (BMSCs). **A**. Scanning electron microscopy was used to characterize the surface of the Ti discs used in this study. The machined-polished Ti discs showed a smooth surface, whereas the Ti disc treated by sand-blasting followed by double acid etching and discrete calcium phosphate nanoparticle deposition (B-DAE-DCD) showed a complex surface topography at the micrometer and nanometer range. **B**. The surface topography was quantitatively evaluated by optical photometry (n = 3 in each group). The B-DAE-DCD surface was approximately 10x rougher than the machined-polished surface. **C**. Human BMSCs cultured on conventional polypropylene culture dishes (n = 4 per time point) or B-DAE-DCD Ti discs (n = 4 per time point) were synchronized by forskolin and exposed to 1 nM 1,25(OH)2D3 vitamin D-supplemented culture medium. The number of BMSCs was determined using a live/dead assay at 24 hours and 72 hours of culture. **D**. Calcein-stained live BMSCs cultured on polypropylene dishes maintained a fibroblastic morphology after 72 hours of culture. By contrast, the live BMSCs on the B-DAE-DCD discs were widely spread and made contacts with adjacent cells, resulting in the establishment of confluency. **E**. The steady state mRNA levels of circadian rhythm-related genes were determined by PCR every 4 hours starting from 24 hours to 72 hours after synchronization (n = 4 per time point in each group). *PER1*, *PER2* and *PER3* from human BMSCs cultured on polypropylene culture dishes and machined-polished discs exhibited a circadian expression pattern. When cultured on the B-DAE-DCD discs, the circadian expression pattern was diminished, while *NPAS2* was upregulated compared to the polypropylene control.

The effect of Ti-based biomaterials on BMSC peripheral circadian rhythms was investigated using commercially pure Ti discs (10 mm or 32 mm in diameter and 1 mm thick). The surface of the Ti discs was left as machined or machined and polished up to 600 grit (machined-polished Ti) (Fig 1A, S1A and S1D Fig). Furthermore, a separate group of Ti disc surfaces was complexed by sandblasting and double-acid etching followed by discrete apposition of hydroxyapatite nanoparticles (B-DAE-DCD) using the production protocol from a commercially available dental implant (T3^®^/NanoTite^™^, Biomet 3I/Zimmer Biomet, Palm Beach Garden, FL) (Fig 1A and 1B). The surface of the Ti discs was characterized by scanning electron microscopy (SEM) and optical photometry (n = 3 in each group). In a comparative study, the cell culture discs were fabricated with zirconia without surface modifications.

### Human BMSC and circadian rhythm gene expression

Immortalized human BMSCs (iMSC3, Applied Biological Materials, Richmond, BC, Canada) were used for this project. Human BMSCs were cultured (20,000 cells per cm^2^) on conventional polypropylene 35-mm culture dishes (n = 4) and B-DAE-DCD discs (35 mm diameter, n = 4) and synchronized with 10 μM forskolin for 2 hours. After extensive washes, human BMSCs were maintained with alpha Minimum Essential Media (MEM), 10% Fetal Bovine Serum (FBS), 1% Penicillin-Streptomycin (PS), and 1 nM 1-alpha, 25-Dihydroxy-Vitamin D3. Twenty-four and seventy-two hours after synchronization, the BMSCs were evaluated with a live/dead assay (Live/Dead^®^ viability/cytotoxicity kit, ThermoFisher Scientific, Canoga Park, CA).

Next, human BMSCs were cultured on polypropylene dishes (n = 4 per time point), machined Ti discs (n = 4 per time point) or B-DAE-DCD discs (n = 4 per time point) as described above. Human BMSCs from each group were harvested every 4 hours starting at 24 hours to 72 hours after the synchronization, and total RNA was prepared. Taqman-based reverse transcription polymerase chain reaction (RT-PCR) was performed in triplicate using commercially available probes for *PER1*, *PER2*, *PER3*, *BMAL1*, *CLOCK* and *NPAS2* and *GAPDH* as an internal control (Life Technologies, Grand Island, NY). The time course PCR data for the circadian rhythm-related gene expression was subjected to cosinor-based rhythmometry analysis [26] using an open source program (http://www.circadian.org/softwar.html) with the period set as 24 hours.

In a separate experiment, human BMSCs were cultured on polypropylene discs (n = 2), B-DAE-DCD discs (n = 2) or zirconia discs (n = 2). RNA samples were harvested at 32 hours after synchronization and subjected to PCR as described above.

### Bone marrow stromal cells (BMSCs) from *Per1*::*luc* rats

Transgenic Wistar rats carrying the luciferase reporter gene sequences in the *Per1* locus (*Per1*::*luc*) [27] were used in this study. Five-month-old male Per1::luc rats (n = 3) underwent euthanasia using 100% CO_2_ gas inhalation. The left and right femurs were removed from each rat, and bone marrow flow through cells were collected using a 20 gauge syringe containing 10 ml Dulbecco's Modified Eagle's medium (DMEM), 10% FBS, and 1% PS. Bone marrow cells were plated onto 85-mm dishes and incubated under 5.0% CO_2_ at 37°C. After two days, the floating cells were removed and the culture medium was replaced with alpha Minimum Essential Medium (alpha MEM) containing 10% FBS and 1% PS. The adherent cells were considered BMSCs. The culture medium was replaced every 2 days until the cells reached 80% confluence. All of the experiments below were performed using the *Per1*:*luc* BMSCs between passages 3 and 5.

In the initial study, forskolin-synchronized BMSCs were cultured with F12 medium containing 10% FBS, 1% PS, and 1 mM luciferin. Time-lapse photomicrographs of reporter-gene expression were generated by a CCD-camera mounted light microscope (Carl Zeiss, Thornwood, NY) at every hour.

### Measurement of the peripheral circadian rhythms in BMSCs using *Per1*::*luc* activity

BMSCs were seeded at 20,000 cells per cm^2^ on 35-mm dishes (n = 4 per experiment) in 2 ml of alpha MEM containing 10% FBS and 1% PS and incubated at 37°C and 5% CO_2_ for 4 days with a medium change. The medium was subsequently changed to 1 ml of alpha MEM containing 10% FBS, 1% PS and 10 μM forskolin and incubated for 2 h. The forskolin-treated BMSCs were washed with phosphate buffered saline (PBS) and cultured in 2 ml F12 medium containing 10% FBS, 1% PS and 1 mM luciferin. The culture dishes were then sealed with high-pressure vacuum grease (Dow Corning Corp., Midland, MI) and concealed from light until they were loaded into the automated high-throughput luminometer (LumiCycle, ActiMetrics, Wilmette, IL). The photon count per second was collected every 10 min from each dish for 5 days. All the raw data were analyzed with the average baseline photon count. The period and amplitude were determined after performing a baseline subtraction with a polynomial filter of 16 and a smoothing of 18 to produce well-defined peaks and troughs from the raw data using a proprietary software program (LumiCycle, ActiMetrics, Wilmette, IL). The “period” was measured from the peak-to-peak x-axis distance over the days while “amplitude” was taken as the peak to trough y-axis distance in photon counts per second. The period and amplitude were compared across the different days and conditions.

### Cell viability and in vitro mineralization of BMSCs cultured on Ti biomaterials under the luminometer measurement conditions

The Ti discs (10 mm diameter) were pre-soaked in alpha MEM overnight. BMSCs (3,000 cells per cm^2^) were then seeded on machined-polished Ti discs, B-DAE-DCD discs and in blank plastic wells and incubated in alpha MEM containing 10% FBS and 1% PS under 5% CO_2_ at 37°C. After 2 days of culture, the cells were washed with PBS and incubated in F12 medium containing 10% FBS, 1% PS and 1 nM vitamin D supplementation. This incubation protocol simulated the luminometry measurement conditions. On culture days 0, 1, 2 and 3, BMSCs on the machined-polished Ti discs, B-DAE-DCD Ti discs or plastic culture wells (n = 3 in each group and at each time point) were subjected to a WST-1 cell viability assay (Clontech Laboratories, Mountain View, CA).

The effects of the Ti substrates on the BMSCs were characterized separately by *in vitro* mineralization assay. BMSCs were plated at 3,000 cells per cm^2^ on 10-mm diameter Ti discs with machined-polished (n = 3) or B-DAE-DCD (n = 3) surfaces that were housed in a 46-well plate. The cells were also cultured in plastic wells (n = 3) without Ti substrates. After the initial seeding, the culture medium was replaced with osteogenic medium containing 1 ml each of dexamethazone (10^−8^), β-glycerophosphate (10 mM), ascorbic acid (50 μg/ml) and 97 ml of alpha MEM, with 10%FBS, 1% PS, and without vitamin D supplementation. The BMSCs were incubated under 5% CO_2_ at 37°C. The different media were changed every three days. On the 13^th^ day, a calcium mineralization assay was performed (Calcium (CPC) LiquiColor test, Stanbio Laboratory, Boerne, TX).

### Measurement of BMSC circadian *Per1*::*luc* expression with Ti substrates

The machined-polished and B-DAE-DCD Ti discs (35 mm in diameter; S2 Fig) were pre-incubated in alpha MEM overnight. BMSCs (20,000 cells per cm^2^) were then seeded in alpha MEM containing 10% FBS and 1% PS. After 4 days, the BMSC were synchronized with forskolin and placed in F12 medium containing 10% FBS, 1% PS and 1 mM luciferin with or without 1 nM vitamin D supplementation. Each group was treated in triplicate, and all of the dishes were prepared for the luminometry measurements. The luminometry measurement of *Per1*::*luc* expression was conducted as described above. The photon count per second was collected every ten minutes from each dish for 5 days, and the data were analyzed as described above. The experiment was repeated at least 3 times and the representative data are presented.

### The effect of vitamin D supplementation and osteogenic medium on BMSC peripheral circadian rhythms

In the vitamin D study, the BMSCs were synchronized by forskolin as mentioned above and cultured in F12 basic medium containing 10% FBS, 1% PS and 1 mM luciferin; these samples were supplemented with 1 nM vitamin D dissolved in ethyl alcohol or the ethyl alcohol vehicle (0.01% final concentration). Separately, forskolin-synchronized BMSCs were cultured in conventional osteogenic medium without or with 1 nM or 10 nM vitamin D supplementation. Each group was treated in triplicate and all the dishes were prepared for the luminometry measurements. The photon count per second was collected every ten minutes from each dish for 5 days, and the data were analyzed as described above.

### Expression of BMSC circadian rhythm genes by reverse transcriptase real-time polymerase chain reaction (RT-PCR)

BMSCs were plated (20,000 cells per cm^2^) on 35 mm dishes with or without B-DAE-DCD discs for 4 days, synchronized by forskolin and cultured in F12 containing 10% FBS, 1% PS with 1 nM vitamin D supplementation. Forty-eight hours later, the total RNA was extracted from the BMSCs (RNeasy Mini Kit, Qiagen, Valencia, CA). Taqman-based RT-PCR was performed in triplicate using commercially available probes for *Per1*, *Per2*, *Clock*, *Bmal1*, *Npas2*, and *Id2* and *Gapdh* as an internal control (Life Technologies, Grand Island, NY).

### Effect of *Npas2* knockdown on circadian rhythm gene expression

To address the effects of *Npas2* on the expression of other circadian rhythm-related genes, an siRNA-derived knockdown study was performed. BMSCs were cultured for 2 days and treated with siRNAs targeting *Npas2* (*NPAS2* siRNA (r), Santa Cruz Biotechnology, Paso Robles, CA) using Lipofectamine according to the manufacturer’s protocol (Life Technologies). For the controls, BMSCs were either untreated or Lipofectamine-treated without the siRNA. siRNA transfection was terminated after 5 hours of incubation and the cells were cultured overnight in fresh medium containing 10% FBS and 1% PS. Then, the BMSCs were forskolin-synchronized and cultured with 1 nM vitamin D supplementation for 32 hours. The preparation of total RNA and Taqman-based RT-PCR for *Per1*, *Per2*, *Bmal1*, and *Clock*, as well as *Gapdh* was performed using commercially available rat probes. RT-PCR was performed in triplicate.

### BMSCs from *Npas2*+/- and *Npas2*-/- mice

To address the effects of *Npas2* on B-DAE-DCD disc-induced circadian rhythm gene suppression, we obtained BMSCs from heterozygous and homozygous mice carrying *Npas2* functional knockdown mutations (B6.129S6-Npas2tm1Slm/J, Jackson Laboratory, Bar Harbor, ME) [28]. Wild-type (C57Bl6, n = 5), *Npas2*+/- (n = 5) and *Npas2*-/- mice (n = 5) were euthanized by 100% CO_2_ gas inhalation, and BMSCs were harvested from their femurs. All the mice were male and between 15 and 20 weeks old. Passage 4 BMSCs (3,000 cells per cm^2^) from each group were cultured on polypropylene dishes, machined Ti discs (n = 2) or B-DAE-DCD discs (10 mm diameter, n = 2), synchronized with forskolin and cultured in F12 containing 10% FBS, 1% PS and 1 nM vitamin D supplementation. RNA samples were harvested 32 hours after synchronization. Expression of the mutant Npas2 gene was evaluated by Taqman-based RT-PCR of *LacZ*, which replaced exon 3 from the Npas2 allele [28]. The expression of the circadian rhythm-related genes was evaluated by Taqman-based RT-PCR using commercially available mouse probes for *Per1*, *Per2*, *Per3*, *Bmal1* and *Clock* and *Gapdh* as an internal control. RT-PCR was performed in duplicate and the mean values are presented.

### Statistical analysis

To evaluate the covariance between the time and amplitudes from the baseline-subtracted data derived from the luminometry experiments, a multivariate repeated measure analysis of variance (MANOVA) was used. This method was also accompanied by the Wilks Lambda test to produce p-values. The amplitudes used in this method were measured by taking the y-axis peak-to-trough amplitude values from days one to four for each treatment condition. The time was measured by taking the midpoint x-axis time value or the half-max between the peak and trough of each amplitude. The sample dish number was equivalent to three or all the treatment groups. The MANOVA analysis was followed by post-hoc evaluation. RT-PCR data of the reference control and the test group were compared by ANOVA and Student’s t test.

### Weighted gene co-expression network analysis (WGCNA)

The whole genome microarray data were obtained from our previous study, which analyzed RNA samples from femur bone marrow tissues exposed to DAE-DCD (S1B and S1C Fig) experimental implants (IT) or osteotomy alone (OS) harvested from vitamin D-sufficient (V+) or -deficient (V-) rats. Hence, the sample groups (n = 4 per group) are designated ITV+, ITV-, OSV+ and OSV-, respectively [20]. The raw microarray data were analyzed using Weighted Gene Co-expression Network Analysis (WGCNA) [29, 30].

Briefly, WGCNA constructs a matrix of pairwise correlations between all pairs of genes across the samples. Biweight midcorrelation was used to minimize the effects of all the possible outliers. A “signed hybrid” network was constructed in which positively correlated genes were connected by strengths that increase with increasing correlation, while negatively correlated genes were considered unconnected. Then, modules were identified using hierarchical clustering followed by branch identification using Dynamic Tree Cut [30]. The module identification procedure resulted in modules containing genes with highly correlated expression profiles. The expression profiles of the genes in each module were summarized using the eigengene; correlation of eigengenes with sample traits were used to quantify the module-trait associations. Two modules (Blue and Turquoise modules) with the strongest eigengene-trait correlation (which also occurred to be the two largest modules) were further analyzed, and functional annotations of the probe IDs were identified using the DAVID (https://david.ncifcrf.gov) online tool.

Genes that were highly correlated with the module eigengene can be considered module hub genes [31, 32]. In particular, we sought to identify the hub genes in the Blue module. The identified hub genes in the Blue module were submitted for STRING protein-protein interaction network analysis (http://string-db.org). Finally, the WGCNA data from the 4 different groups were reorganized for OSV+ and ITV+ as well as ITV+ and ITV- to dissect the roles of the Ti biomaterials and vitamin D in the gene network formation.

## Results

### Ti biomaterials

In this study, we designed Ti disc substrates suitable for BMSC culture that received one of the following surface treatments: (1) machined and polished up to 600-grid (machined-polished) to remove major macro- to micro- surface topography elements; (2) machined without polishing; or (3) sand blasted and double acid etched, followed by a discrete modification at the nanometer level by chemical bonding of hydroxyapatite nanoparticles (B-DAE-DCD) to contribute to the increased micro- and nanotopography (Fig 1A and 1B and S1E and S1F Fig). Forskolin-synchronized human BMSCs cultured on B-DAE-DCD discs were compared to those cultured on conventional polypropylene culture dishes. After 24 hours, a live/dead assay suggested that the number of live BMSCs was equivalent in both groups. However, after 72 hours, the number of live BMSCs on the polypropylene dish was notably higher than on the B-DAE-DCD discs (Fig 1C). The different BMSC behaviors were not explained by the decreased BMSC viability. The calcein-positive live BMSCs on the polypropylene dish exhibited fibroblastic morphologies. In contrast, the cells on the B-DAE-DCD discs were spread widely and made contact with adjacent cells (Fig 1D). The BMSCs on the B-DAE-DCD discs apparently reached a confluent state, which might have contributed to the slowed increase in cell number. Our data were consistent with previously established differential BMSC behaviors on Ti biomaterials with complex surfaces [33–35].

### Expression of circadian rhythm-related genes by human BMSCs cultured on Ti biomaterials

Human BMSCs were forskolin-synchronized and cultured on polypropylene dishes, machine-polished Ti discs or B-DAE-DCD discs with vitamin D supplementation. BMSCs cultured on the polypropylene dishes and the machined-polished discs demonstrated normal *PER1*, *PER2* and *PER3* expression circadian patterns (Fig 1E and S1 Table). However, when the BMSCs were cultured on the B-DAE-DCD discs, *PER1*, *PER2*, and *PER3* expression appeared to be decreased (Fig 1E) and the acrophase was extended (S1 Table). Separately, a striking overexpression of *NPAS2* was also observed (Fig 1E).

### Ti substrates suppressed *Per1*::*luc* circadian expression and increased *Npas2* expression in rat BMSCs

**Fig 2:**
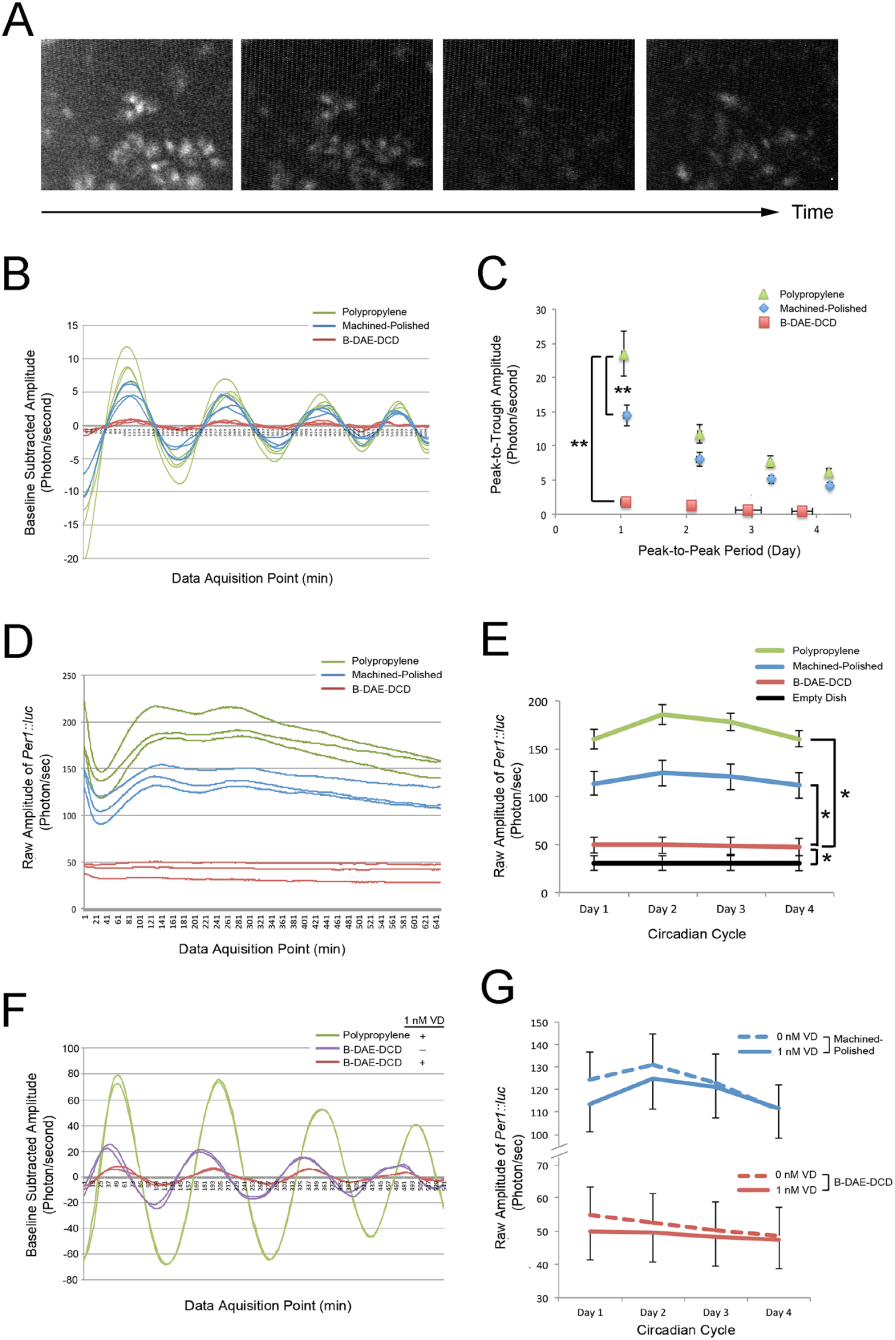
Circadian expression of the *Per1*::*luc* reporter gene in BMSCs. Femur-derived BMSCs were harvested from 3 male *Per1*::*luc* rats (5 months old). BMSCs from passages 3 to 5 were used for the experiments. **A**. Time-lapse microscopy depicted the periodic activation of *Per1*::*luc* reporter gene expression in the BMSCs. **B**. *Per1*::*luc* reporter gene expression was measured by luminometry. The experiment was performed with three dishes in each group. The baseline-subtracted luminometry data demonstrated the circadian activation of *Per1*::*luc* expression when BMSCs were cultured on polypropylene dishes with 1 nM vitamin D supplementation; this was reduced on the Ti discs. Strikingly, the circadian rhythm of *Per1*::*luc* expression was nearly completely lost on the B-DAE-DCD discs. **C**. The loss of *Per1*::*luc* circadian expression on the B-DAE-DCD discs was depicted by the decreased peak-to-trough amplitude and peak-to-peak period. **: p<0.01 by Wilks Lambda for the amplitude and period. **D**. The raw amplitude measurements depicted the stable expression of the *Per1*::*luc* reporter gene after the initial forskolin shock when the BMSCs were cultured on the polypropylene dishes or machined-polished Ti discs. In contrast, *Per1*::*luc* reporter gene expression remained low when the BMSCs were cultured on the B-DAE-DCD discs. **E**. The average raw amplitude of the *Per1*::*luc* reporter gene was significantly lower in the B-DAE-DCD group than the polypropylene and machine-polished groups; however, it was above the background level of the no cell negative control. **: p<0.01 by ANOVA. **F**. When BMSCs were cultured on B-DAE-DCD Ti discs without vitamin D supplementation, the lost *Per1*::*luc* circadian expression was partially recovered. **G**. The raw amplitudes from the luminometry data indicated that vitamin D supplementation did not affect Per1 expression levels in BMSCs cultured on the machined-polished Ti discs or B-DAE-DCD discs.

This study further employed BMSCs derived from transgenic rats carrying the *Per1*::*luc* allele. The SCN from this rat model sustained *ex vivo* circadian expression of *Per1*::*luc* over an extended culture period [27], while peripheral tissues such as skeletal muscle, lung and liver demonstrated periodic *Per1*::*luc* expression after forskolin-synchronization [36]. Forskolin-synchronized femur BMSCs demonstrated circadian *Per1*::*luc* expression detected by time-lapse microscopy (Fig 2A). The baseline-subtracted luminometry data showed the highly regulated circadian expression of *Per1*::*luc* in BMSCs when cultured on the polypropylene dishes (Fig 2B). The peak-to-trough amplitude showed a progressive decrease during the culture period, whereas the peak-to-peak circadian duration was maintained at approximately 24 hours for 3 to 4 days of culture (Fig 2C).

When the BMSCs were cultured on Ti substrates with vitamin D supplementation, the baseline-subtracted data indicated a significant change in the *Per1*::*luc* circadian rhythm (Fig 2B). BMSCs on the machined-polished Ti substrate maintained their circadian rhythm, which was suggested by the consistent peak-to-peak periods, whereas the peak-to-trough amplitude was significantly reduced (Fig 2C). The effect of the B-DAE-DCD substrate was striking and showed that *Per1*::*luc* circadian expression was almost completely abrogated (Fig 2B and 2C).

The raw luminometry data further indicated that *Per1*::*luc* expression was significantly downregulated in the BMSCs cultured on the Ti substrates (Fig 2D). In particular, the B-DAE-DCD substrate decreased *Per1*::*luc* expression near to the measurement limit of an empty dish (Fig 2E). Next, we examined whether the loss of *Per1*::*luc* expression was due to a loss in BMSC viability. The rat BMSCs cultured in the sealed culture dishes with or without Ti substrates maintained similar viabilities (S2 and S3 Figs). Therefore, the downregulation of *Per1*::*luc* was not due to a loss of cell viability and must be due to a previously unrecognized effect of Ti biomaterials.

### *Per1*::*luc* BMSCs demonstrated peripheral circadian rhythm plasticity with vitamin D supplementation and osteogenic medium

**Fig 3:**
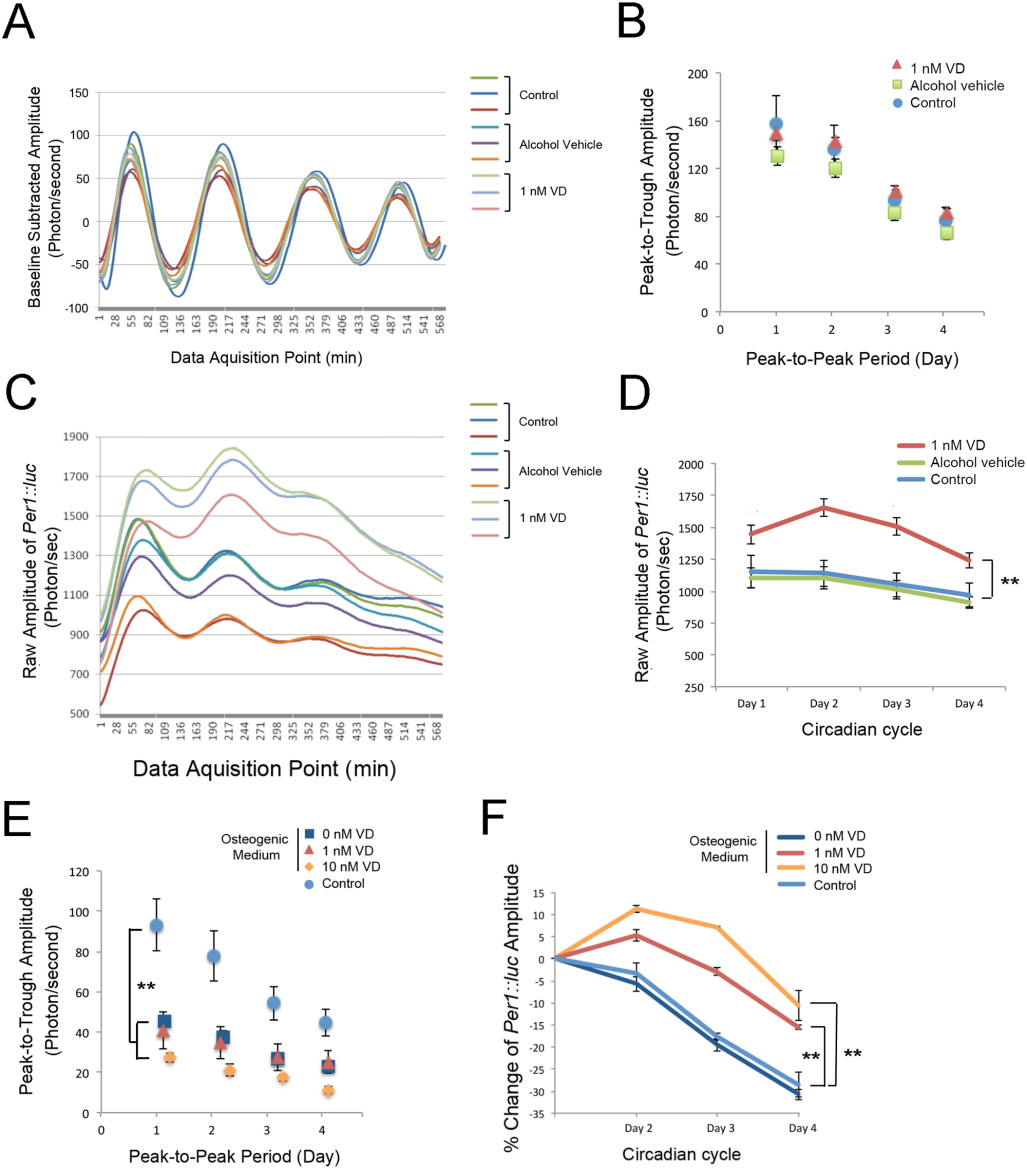
Characterization of the *Per1*::*luc* circadian rhythm in BMSCs. All the luminometry experiments were performed in triplicate for each group. **A**. The baseline-subtracted luminometry data demonstrated the steady circadian expression of *Per1*::*luc* of BMSC, which progressively decreased over the culture period. **B**. The average peak-to-trough amplitude and peak-to-peak period by day showed the tightly regulated *Per1*::*luc* circadian expression, which was not affected by 1 nM vitamin D supplementation. **C**. The raw amplitude data suggested that there was the steady decline in BMSC *Per1*::*luc* expression over the culture period after the initial forskolin shock. **D**. BMSC vitamin D supplementation was found to increase *Per1*::*luc* expression. **: p<0.01 by ANOVA. **E**. When BMSCs were exposed to osteogenic differentiation medium, both the baseline-subtracted peak-to-trough amplitude and the peak-to-peak period were modulated. Vitamin D supplementation appeared to accelerate the effects of osteogenic medium. **: p<0.01 by Wilks Lambda ANOVA for the amplitude and period. **F**. Vitamin D supplementation with osteogenic medium sustained *Per1*::*luc* expression throughout the culture period. **: p<0.01 by ANOVA.

Ti biomaterials induce osteogenic differentiation during osseointegration [37, 38], while vitamin D deficiency promotes negative effects. Therefore, in this project, we investigated the effects of vitamin D and osteogenic medium supplementation [39] on BMSC circadian rhythm plasticity. The lack of vitamin D supplementation in the culture medium appeared to partially recover the circadian expression of *Per1*::*luc* that was suppressed by the B-DAE-DCD substrate (Fig 2F and 2G). Vitamin D supplementation without Ti substrates had little effect on the peak-to-peak period and peak-to-trough amplitude of circadian *Per1*::*luc* expression (Fig 3A and 3B); however, the raw luminometry data revealed significantly increased *Per1*::*luc* expression with vitamin D supplementation (Fig 3C and 3D).

The osteogenic medium did not affect the peak-to-peak period, though it significantly decreased the peak-to-trough amplitude (Fig 3E). The *Per1*::*luc* expression pattern in osteogenic medium with vitamin D supplementation was similar to that of vitamin D supplementation alone and was sustained throughout the experimental period (Fig 3F).

### The role of *Npas2* overexpression in the aberrant circadian rhythm

**Fig 4:**
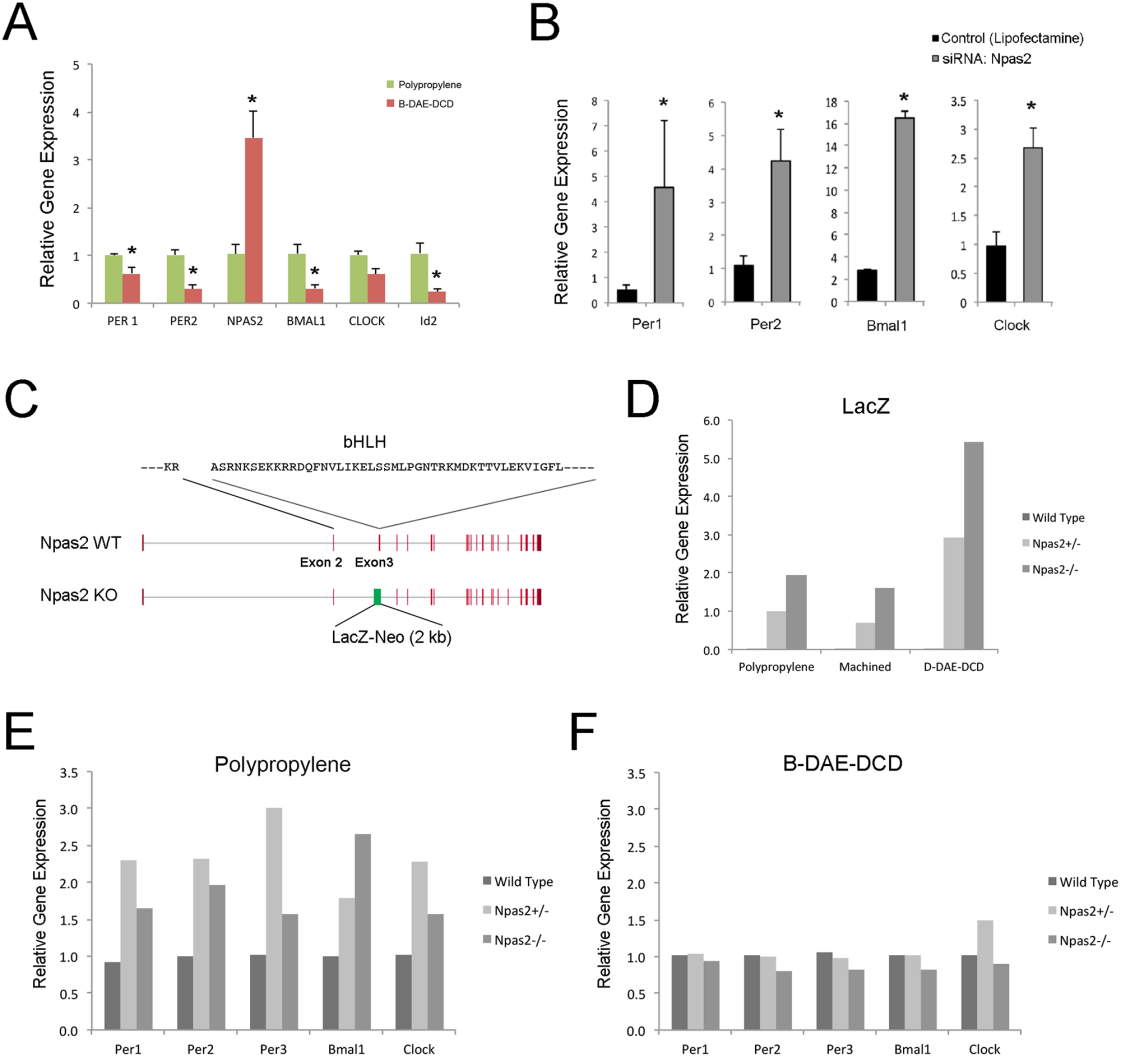
The role of *Npas2* in BMSC circadian rhythm. **A**. A snapshot of circadian rhythm gene expression from rat BMSCs shows the transcriptional downregulation of *Per1*, *Per2*, *Bmal1* and *Id2* when the BMSCs were cultured on B-DAE-DCD Ti discs. A similar upregulation in *Npas2* was observed in human BMSCs. RT-PCR was performed in triplicates. *: p<0.05 by Student’s t-test. **B**. The role of the transcription factor *Npas2* on the steady state mRNA levels of circadian rhythm-related genes was examined by siRNA. *Npas2* knock down appeared to have a greater impact on circadian rhythm-related gene expression in BMSCs cultured on the polypropylene dishes. *: p<0.05 by Student’s t-test. **C**. To further clarify the role of *Npas2*, BMSCs were harvested from mice carrying the *Npas2* allele lacking Exon 3, which was replaced by the *LacZ* reporter gene cassette, resulting in a *Npas2* functional knockout mutation due to the lack of the basic helical-loop-helical (bHLH) domain. **D**. BMSCs were harvested from the femurs of wild-type, *Npas2*+/- and *Npas2*-/- male mice (n = 5 each). BMSCs (passage 4) were cultured and synchronized on polypropylene dishes, machined Ti discs or B-DAE-DCD discs (n = 2 in each group). Thirty-two hours after synchronization, *LacZ* reporter gene expression was increased in the D-DAE-DCD disc group. **E**. The expression of circadian rhythm genes were observed to increase in the *Npas2*+/- and *Npas2*-/- BMSCs maintained on polypropylene culture dishes for 32 hours after synchronization. **F**. *Npas2*+/- and *Npas2*-/- BMSC maintained on B-DAE-DCD discs for the same length of time demonstrated similar circadian rhythm gene expression patterns as wild-type mouse BMSCs.

Rat BMSCs cultured on polypropylene dishes or B-DAE-DCD discs were collected at 48 hours for Taqman-based RT-PCR assessment. BMSC cultured on the B-DAE-DCD discs revealed the downregulation of circadian rhythm-related genes containing E-box elements in their promoters (*Per1*, *Per2*, *Bmal1*, and *Id2*), whereas *Npas2* alone was significantly upregulated (Fig 4A). Using the RT-PCR data from the untreated BMSCs as a reference, siRNA-derived knock down of *Npas2* resulted in a universal increase in *Per1*, *Per2*, *Bmal1* and *Clock* expression when the BMSCs were cultured on polypropylene dishes (Fig 4B).

The role of *Npas2* in the aberrant BMSC circadian rhythm was further investigated using BMSCs isolated from *Npas2* functional knockout mice. In this mouse line, Exon 3, which encodes the basic-helix-loop-helix domain, was replaced by a *LacZ* reporter gene cassette (Fig 4C). *LacZ* expression increased when *Npas2*+/- and *Npas2*-/- BMSCs were cultured on B-DAE-DCD discs (Fig 4D). BMSCs with the *Npas2* knockout mutation cultured on polypropylene dishes exhibited increased *Per1*, *Per2*, *Per3*, *Bmal1* and *Clock* expression (Fig 4E). This expression pattern in the *Npas2* knockout BMSCs was consistent with rat BMSCs treated with *Npas2* siRNA. However, to our surprise, the expression levels of the circadian rhythm-related genes was unaffected when *Npas2*+/- and *Npas2*-/- BMSCs were cultured on the B-DAE-DCD discs (Fig 4F). Therefore, it is unlikely that Ti biomaterial-induced overexpression of *Npas2* caused the aberrant circadian rhythms observed when the BMSCs were exposed to the B-DAE-DCD discs.

### Weighted gene co-expression network analysis (WGCNA) of the in vivo microarray data derived from rat femur bone marrow tissue associated with the DAE-DCD Ti implant and vitamin D deficiency

**Fig 5:**
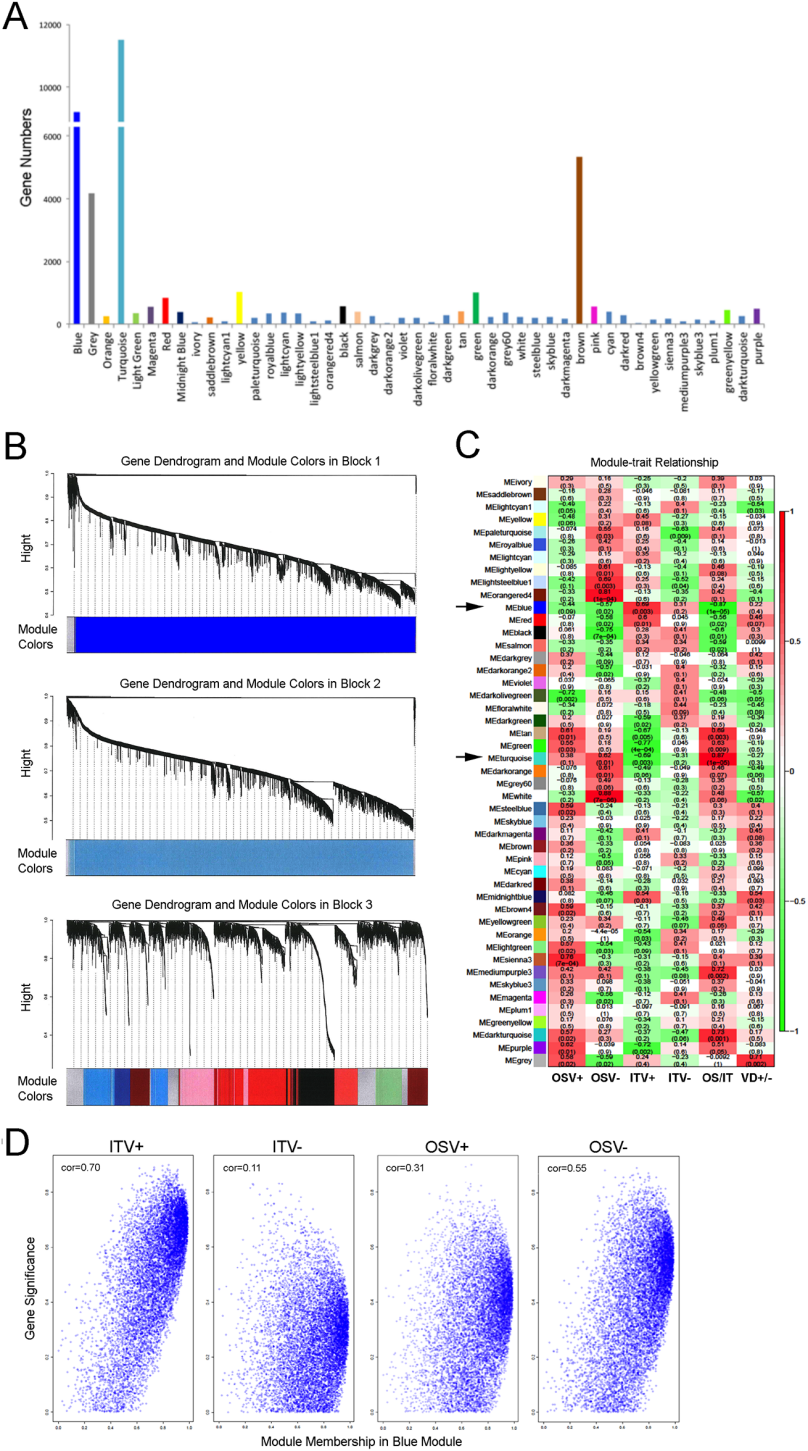
The weighted gene co-expression network analysis (WGCNA). The microarray data were obtained previously from rat femur bone marrow tissue after osteotomy surgery with or without DAE-DCD Ti implants with sufficient or deficient serum vitamin D levels (ITV+, ITV-, OSV+ and OSV- groups, respectively) [20]. **A**. WGCNA identified 47 modules of co-expressed genes. The Turquoise and Blue modules contained a disproportionately large number of genes. **B**. A dendrogram of the WGCNA analysis suggested an unusually large co-expressed gene network of hierarchical clustering among Blue and Turquoise modules, which contained 9,202 and 11,511 genes, respectively. **C**. Association of the module eigengenes with experimental bone marrow tissue traits responding to osteotomy surgery with or without Ti implant placement (IT and OS, respectively) in vitamin D-sufficient and -deficient rats (V+ and V-, respectively). Out of the 47 modules identified, the Blue and Turquoise modules (arrows) showed the highest eigengene correlation in OS/IT. Within each cell, upper values indicate correlation coefficients between the module eigengene and the traits, while the lower values indicate the corresponding p-value. **D**. Scatter plots of module membership (eigengene-based connectivity) and gene significance for the Blue module for each of the therapeutic traits (ITV+, ITV-, OSV+ and OSV-). The highest correlation between module membership and gene significance was in ITV+; this was noticeably decreased in ITV-.

To address the differential role of the peripheral circadian rhythm during the osseointegration of Ti biomaterials, we analyzed the available microarray data obtained from our previous study [20] using rat femur tissues adjacent to experimental Ti implants with a DAE-DCD surface (S1B and S1C Fig). The microarray expression data were obtained from the following 4 independent rat groups (n = 4 in each group): (1) OSV+, femur osteotomy wound healing in a vitamin D-sufficient rat; (2) OSV-, femur osteotomy wound healing in a vitamin D-deficient rat; (3) ITV+, femur tissue around a DAE-DCD implant in a vitamin D-sufficient rat; and (4) ITV-, femur tissue around a DAE-DCD implant in a vitamin D-deficient rat. WGCNA identified 47 modules; for convenient visualization, each module was assigned a colored label. Among the modules, the Blue and Turquoise modules were the largest modules (9,202 and 11,511 genes, respectively: Fig 5A) suggesting the highly organized gene co-expression networks in Blocks 1 and 2, respectively (Fig 5B).

The probe IDs from each module were exported to the DAVID online tool [40], which revealed vitamin D receptor (*Vdr*) and eight circadian rhythm-related genes, including *Npas2*, *Bmal1*, *Clock*, two casein kinase subtypes and three other helix-loop-helix family member subtypes, in the Blue module. *Per1*, *Per2* and *Per3*, which are negative-elements in the mammalian circadian molecular clock feedback loop, were found in the Turquoise module. The Blue and Turquoise modules exhibited the highest eigengene correlation of 0.87 (p = 0.00005) when comparing the osteotomy without and with implant placement (OS/IT) (Fig 5C). Furthermore, the Blue and Turquoise modules changed in the opposite direction with strong eigengene correlations for each of the different traits. A scatter plot between gene significance and module membership in the Blue module showed that the module hub genes tended to also have the strongest association with ITV (Fig 5D).

**Fig 6:**
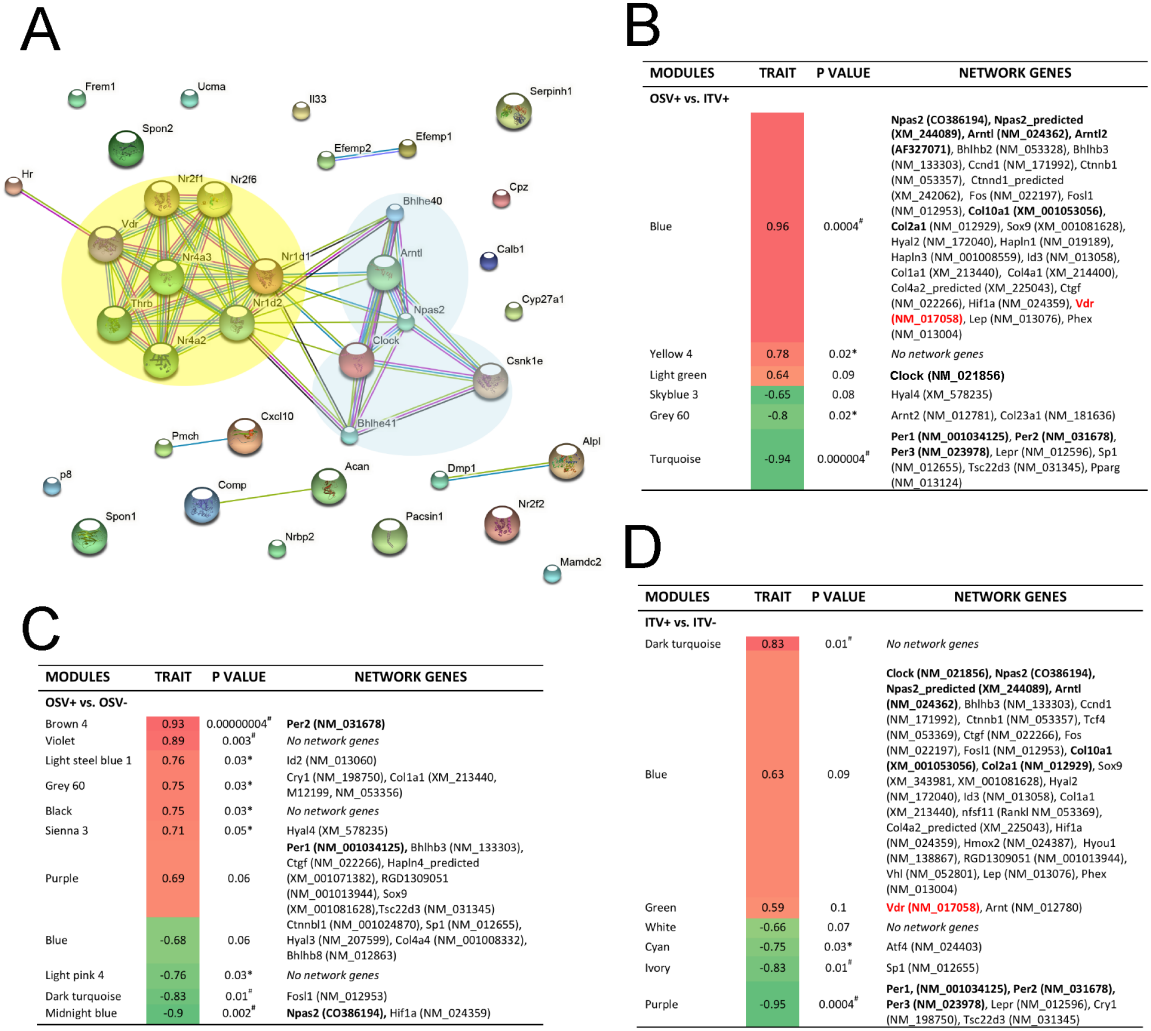
Gene networks formed around the biomaterial implant. **A**. The protein-protein interaction network of a total of 36 unique hub genes from the Blue module suggested interacting core networks between circadian rhythm/E-box binding proteins and nuclear steroid hormone receptors, including the vitamin D receptor (*Vdr*). **B**. The interaction between circadian rhythm-related genes and connective tissue extracellular matrix genes in the comparison between OSV+ and ITV+ indicated that the trait-significant Blue module contained *Npas2*, *Bmal1* (*Arntl*) and *Vdr*, whereas the Turquoise module contained *Per1*, *Per3* and *Per3*. **C**. The comparison between OSV+ and OSV- without implanted biomaterials did not detect *Vdr* and *Npas2* was found to be significantly downregulated. **D**. The comparison between ITV+ and ITV- revealed that the Blue module lost its trait-significance and Vdr. *Per1*, *Per2* and *Per3* were also moved from the Turquoise module and to the Purple module.

These analyses suggested that the Blue module contains the trait modulating gene network; thus, we examined the gene network and identified hub genes in the Blue module. Comparative evaluation of Blue module between the ITV+ and ITV- samples was thought to provide clues to understanding the role of vitamin D in bone marrow tissue exposed to Ti biomaterials. We then established the node number of each gene and selected those above the median nod number, resulting in 37 unique genes (S2 Table). Kyoto Encyclopedia of Genes and Genomes analysis (S3 Table) and Gene Ontology analysis (S4 Table) identified circadian rhythm, steroid hormone receptor, and vitamin D binding pathways. The identified hub genes from the Blue module were submitted to search for functional protein association networks, which suggested that the circadian rhythm/E-box binding network and nuclear steroid hormone receptors (including *Vdr*, *Rev-ErbA* and *Rev-ErbA-beta*) would interact (Fig 6A).

Finally, the effect of the Ti biomaterial (DAE-DCD) was evaluated by comparing OSV+ and ITV+, which formed the trait-specific upregulated Blue module containing *Npas2* and *Bmal1* (*Arntl*) as well as *Vdr*. The downregulated Turquoise module was also trait-specific and contained *Per1*, *Per2* and *Per3* (Fig 6B and S5 Table). Interestingly, *Clock* was found in the trait-neutral Light green module. When ITV+ and ITV- were compared, the Blue module, which contained *Npas2*, *Bmal1* and *Clock*, was no longer trait-specific. *Vdr* was not found in any network when OSV+ and OSV- were compared (Fig 6C) and was found in the trait-neutral Green module (Fig 6D and S6 Table). The downregulation of *Per1*, *Per2* and *Per3* sustained and formed part of the Purple module.

## Discussion

This study demonstrated highly unusual peripheral circadian rhythms of BMSCs induced by Ti biomaterials with complex surfaces at an unprecedented level. BMSCs are the major cellular component in the bone marrow, which contains mesenchymal stem cells, and are capable of differentiating in multiple lineages and centrally coordinating bone remodeling and regeneration [41]. However, how BMSCs respond to a wide range of environmental cues that occur during fracture wound healing, bone marrow ablation or surgical implant placement has not been fully investigated. This study demonstrated that the peripheral circadian rhythm of BMSCs was modulated by Ti biomaterials with complex surfaces at unprecedented levels (Fig 1). BMSCs almost completely abrogated the circadian expression of *Per1*, particularly those cultured on Ti substrates with complex surface modifications (Fig 2). Increasing numbers of reports suggest that bone tissues possess an independent peripheral circadian clock mechanism. Chen et al. (2000) demonstrated for the first time the expression profile of *Per* genes in murine bone marrow in a lineage- and/or differentiation stage-dependent manner [42]. Zvonic et al. (2007) reported the oscillatory expression of the core circadian rhythm genes in the mouse calvarial bone, and further showed that over 20% of the genes expressed in the calvarial bone also followed the oscillatory profile [43]. Zhang et al. (2008) showed the E-box-related regulation of osteoblast differentiation in MC3T3-E1 cells by multiple Helix-Loop-Helix (HLH) factors, such as *Id-2*, *Id-3* and *Id-4*, which can functionally regulate the expression of bone markers [44]. McElderry et al. (2013) demonstrated a burst in active mineral deposition in calvarial organ culture followed by a decreased or quiescent phase with a periodicity of approximately 27 hours without central SCN control [45].

The significance of the present study lies in the demonstration that exogenous stimuli derived from commonly used Ti biomaterials with complex surfaces significantly disrupted the circadian rhythm. The therapeutic outcome of a successful endosseous implant is achieved, in part, by the osseointegration of Ti biomaterials as well as other materials, such as zirconia. We further investigated the circadian rhythm by examining gene expression on zirconia without surface modifications. Machined Ti discs and smooth surface zirconia discs induced similar circadian rhythm gene expression patterns, whereas the B-DAE-DCD discs showed robust *Npas2* expression (S4 Fig). Therefore, the complex surface modifications, not the materials’ chemistry, may play a significant role in circadian rhythm modulation.

It has been shown that serum vitamin D deficiency may negatively affect the initial establishment of osseointegration [46, 47]. The present study revealed the distinct effect of vitamin D supplementation on circadian Per1 expression in BMSCs (Fig 3). The intrinsic circadian clock is maintained by a network of core molecular components [48], as well as by increasing numbers of clock-modifying molecules [49]. The WGCNA evaluation of the whole genome microarray data identified large gene co-expression modules containing circadian rhythm transcription factors (Blue module) and repressor proteins (Turquoise module), both of which are sensitively associated with rat bone marrow tissue treatment traits for exposure to Ti biomaterials or serum vitamin D levels (Fig 5). The hub genes in the Blue module suggested that the circadian rhythm core molecular components and steroid hormone nuclear receptors interacting protein networks include *Vdr*. When the gene network modules were further dissected into ITV+ and ITV-, the circadian rhythm gene network in the Blue module appeared to deteriorate under the vitamin D-deficient conditions (Fig 6). *Vdr* has been identified as a circadian clock modifier in adipose tissue [50], and vitamin D supplementation alone has been reported to synchronize adipose-derived adult mesenchymal stem cells (ADSC) [51]. The genomic and non-genomic actions of vitamin D on bHLH genes may explain the secondary effects on these genes, including fine tuning the circadian rhythms [52]. The present study further suggests that *Vdr* in BMSCs may play an essential role in organizing the Ti biomaterial-activated aberrant circadian rhythm.

It was reported that *Npas2* expression was not detected in bone but that its deletion did not affect bone mass [53]. Consistently, BMSCs in the present study showed only baseline expression of *Npas2* when cultured on conventional plates without Ti discs. Strikingly, this study identified an unusual upregulation of *Npas2* in BMSCs exposed to Ti biomaterials with complex surface topographies (Figs 1 and 2). *Npas2* has overlapping functions with *Clock* and forms a heterodimer with *Bmal1*, which functions as an E-box binding enhancer for *Per* and *Cry* homologues [54] as well as for other genes that contain E-box elements, such as *Id2*, c-myc, collagens type I, II and X [55]. Thus, the abnormally increased *Npas2* expression was initially thought to contribute to the aberrant circadian rhythm patterns observed in the BMSCs induced by Ti biomaterials with complex surfaces. However, examination of BMSCs from *Npas2* knockout mice did not support this hypothesis (Fig 4).

Endogenous agents are involved in the response of clock molecules to different environmental cues [56], such as glucose feeding, light response, serum shock, and glucocorticoid and hormone exposure [57–59]. The surface of Ti biomaterials is spontaneously oxidized to form a native layer of TiO_2_, which may represent an environmental cue for bone cell plasticity. Surface treatments, such as acid etching [60] and hydroxyapatite nanocoatings [61], significantly alter the surface oxide layer. Recently, thermal oxidation of Ti biomaterials was reported to increase the TiO_2_ surface layer, resulting in increased biological activity [62]. The interaction between the endogenous molecule heme and TiO_2_ has been described [63, 64]. It has also been reported that *Npas2*, which contains heme prosthetic groups, functions as a gas-sensor [65]. Mutation of the heme domain and its conformation changes reduce *Per1* transcription because the mutational and conformational changes impair *Bmal1* and *Npas2* heterodimer formation, resulting in a loss of DNA binding to the canonical E-box regulatory sequence in various target genes [42, 66]. It is tempting to speculate that *Npas2* upregulation in BMSCs might be induced by the oxidation levels on the surface of the Ti biomaterials, which may initiate titanium actions at the local bone site by activating gas-sensor or heme metabolism pathways.

In conclusion, this study postulates that the significant degree of aberrant plasticity demonstrated by the peripheral circadian clock of BMSCs exposed to Ti biomaterials may provide the ability to integrate environmental clues and therefore to coordinate a coherent biological output. The data in this study corroborate previous *in vivo* and *in vitro* findings (Mengatto et al., 2011) and highlight the increase in *Npas2* and *Per1* abrogation as independent key factors for osseointegration. These results have potential practical implications related to orthopedic and dental implant patients, as understanding the molecular and cellular mechanisms during early titanium implant osseointegration allows for the development of better biomarkers to follow up healing cellular processes. This approach also enables the therapeutic conditioning of local bone or systemic situations to achieve faster and more efficient responses from BMSCs during the integration of Ti biomaterials into bone.

## Supporting information

S1 Fig Surface characterization of Ti substrates by SEM. (PDF)

S2 Fig Ti discs fit in 35 mm culture dish used for this study. (PDF)

S3 Fig The cell viability test of BMSC in the luminometer culture condition. (PDF)

S4 Fig Circadian rhythm-related gene expression by BMSC cultured on Ti discs with machined or B-DAE-DCD surface, as well as Zirconia disc. (PDF)

S1 Table Cosinor-based rhythmometry analysis of circadian rhythm gene expression in human BMSC cultured on polypropylene dish, machined Ti disc or B-DAE-DCD disc (24h fixed cosine curve). (PDF)

S2 Table Hub genes in Blue module. (PDF)

S3 Table Kyoto Encyclopedia of Genes and Genomes (KEGG) analysis of Blue module hub genes. (PDF)

S4 Table Gene ontogeny analysis (Function) of Blue module hub genes. (PDF)

S5 Table ITV+ vs. OSV+ trait comparison. (PDF)

S6 Table ITV+ vs. ITV- trait comparison. (PDF)

## References

[1] McAfee PC, Farey ID, Sutterlin CE, Gurr KR, Warden KE, Cunningham BW. The effect of spinal implant rigidity on vertebral bone density. A canine model. Spine (Phila Pa 1976). 1991;16(6 Suppl):S190–7. . 1862413 10.1097/00007632-199106001-00003

[2] Long M, Rack HJ. Titanium alloys in total joint replacement—a materials science perspective. Biomaterials. 1998;19(18):1621–39. . 9839998 10.1016/s0142-9612(97)00146-4

[3] Abdel-Hady Gepreel M, Niinomi M. Biocompatibility of Ti-alloys for long-term implantation. J Mech Behav Biomed Mater. 2013;20:407–15. doi: 10.1016/j.jmbbm.2012.11.014 . 23507261 10.1016/j.jmbbm.2012.11.014

[4] Davies JE. Understanding peri-implant endosseous healing. J Dent Educ. 2003;67(8):932–49. . 12959168

[5] Veronesi F, Giavaresi G, Fini M, Longo G, Ioannidu CA, Scotto d'Abusco A, et al Osseointegration is improved by coating titanium implants with a nanostructured thin film with titanium carbide and titanium oxides clustered around graphitic carbon. Mater Sci Eng C Mater Biol Appl. 2017;70(Pt 1):264–71. doi: 10.1016/j.msec.2016.08.076 . 27770890 10.1016/j.msec.2016.08.076

[6] Smeets R, Stadlinger B, Schwarz F, Beck-Broichsitter B, Jung O, Precht C, et al Impact of Dental Implant Surface Modifications on Osseointegration. Biomed Res Int. 2016;2016:6285620 doi: 10.1155/2016/6285620 ; 27478833 10.1155/2016/6285620 PMC4958483

[7] Rao PJ, Pelletier MH, Walsh WR, Mobbs RJ. Spine interbody implants: material selection and modification, functionalization and bioactivation of surfaces to improve osseointegration. Orthop Surg. 2014;6(2):81–9. doi: 10.1111/os.12098 . 24890288 10.1111/os.12098 PMC6583242

[8] Kulkarni M, Mazare A, Gongadze E, Perutkova S, Kralj-Iglic V, Milosev I, et al Titanium nanostructures for biomedical applications. Nanotechnology. 2015;26(6):062002 doi: 10.1088/0957-4484/26/6/062002 . 25611515 10.1088/0957-4484/26/6/062002

[9] Tomsia AP, Lee JS, Wegst UG, Saiz E. Nanotechnology for dental implants. Int J Oral Maxillofac Implants. 2013;28(6):e535–46. doi: 10.11607/jomi.te34 . 24278949 10.11607/jomi.te34

[10] Nishimura I, Huang Y, Frank Butz F, Ogawa T, Lin A, Wang CJ. Discrete deposition of hydroxyapatite nanoparticles on a titanium implant with predisposing substrate microtopography accelerated osseointegration. Nanotechnology. 2007;18(24):245101.

[11] Kagan R, Adams J, Schulman C, Laursen R, Espana K, Yoo J, et al What Factors Are Associated With Failure of Compressive Osseointegration Fixation? Clin Orthop Relat Res. 2016 doi: 10.1007/s11999-016-4764-9 . 26926774 10.1007/s11999-016-4764-9 PMC5289163

[12] Jimbo R, Albrektsson T. Long-term clinical success of minimally and moderately rough oral implants: a review of 71 studies with 5 years or more of follow-up. Implant Dent. 2015;24(1):62–9. doi: 10.1097/ID.0000000000000205 . 25621551 10.1097/ID.0000000000000205

[13] Oduwole KO, Molony DC, Walls RJ, Bashir SP, Mulhall KJ. Increasing financial burden of revision total knee arthroplasty. Knee Surg Sports Traumatol Arthrosc. 2010;18(7):945–8. doi: 10.1007/s00167-010-1074-8 . 20148322 10.1007/s00167-010-1074-8

[14] Kelly J, Lin A, Wang CJ, Park S, Nishimura I. Vitamin D and bone physiology: demonstration of vitamin D deficiency in an implant osseointegration rat model. J Prosthodont. 2009;18(6):473–8. doi: 10.1111/j.1532-849X.2009.00446.x . 19486459 10.1111/j.1532-849X.2009.00446.x

[15] Dvorak G, Fugl A, Watzek G, Tangl S, Pokorny P, Gruber R. Impact of dietary vitamin D on osseointegration in the ovariectomized rat. Clin Oral Implants Res. 2012;23(11):1308–13. doi: 10.1111/j.1600-0501.2011.02346.x . 22151621 10.1111/j.1600-0501.2011.02346.x

[16] Cho YJ, Heo SJ, Koak JY, Kim SK, Lee SJ, Lee JH. Promotion of osseointegration of anodized titanium implants with a 1alpha,25-dihydroxyvitamin D3 submicron particle coating. Int J Oral Maxillofac Implants. 2011;26(6):1225–32. . 22167427

[17] Wu YY, Yu T, Yang XY, Li F, Ma L, Yang Y, et al Vitamin D3 and insulin combined treatment promotes titanium implant osseointegration in diabetes mellitus rats. Bone. 2013;52(1):1–8. doi: 10.1016/j.bone.2012.09.005 . 22985888 10.1016/j.bone.2012.09.005

[18] Zhou C, Li Y, Wang X, Shui X, Hu J. 1,25Dihydroxy vitamin D(3) improves titanium implant osseointegration in osteoporotic rats. Oral Surg Oral Med Oral Pathol Oral Radiol. 2012;114(5 Suppl):S174–8. doi: 10.1016/j.oooo.2011.09.030 . 23063395 10.1016/j.oooo.2011.09.030

[19] Liu W, Zhang S, Zhao D, Zou H, Sun N, Liang X, et al Vitamin D supplementation enhances the fixation of titanium implants in chronic kidney disease mice. PLoS One. 2014;9(4):e95689 doi: 10.1371/journal.pone.0095689 ; 24752599 10.1371/journal.pone.0095689 PMC3994107

[20] Mengatto CM, Mussano F, Honda Y, Colwell CS, Nishimura I. Circadian rhythm and cartilage extracellular matrix genes in osseointegration: a genome-wide screening of implant failure by vitamin D deficiency. PLoS One. 2011;6(1):e15848 doi: 10.1371/journal.pone.0015848 ; 21264318 10.1371/journal.pone.0015848 PMC3019224

[21] Konopka RJ, Hamblen-Coyle MJ, Jamison CF, Hall JC. An ultrashort clock mutation at the period locus of Drosophila melanogaster that reveals some new features of the fly's circadian system. J Biol Rhythms. 1994;9(3–4):189–216. doi: 10.1177/074873049400900303 . 7772790 10.1177/074873049400900303

[22] Rothenfluh A, Young MW, Saez L. A TIMELESS-independent function for PERIOD proteins in the Drosophila clock. Neuron. 2000;26(2):505–14. . 10839368 10.1016/s0896-6273(00)81182-4

[23] Takahashi JS, Hong HK, Ko CH, McDearmon EL. The genetics of mammalian circadian order and disorder: implications for physiology and disease. Nat Rev Genet. 2008;9(10):764–75. doi: 10.1038/nrg2430 ; 18802415 10.1038/nrg2430 PMC3758473

[24] Takahashi JS, Shimomura K, Kumar V. Searching for genes underlying behavior: lessons from circadian rhythms. Science. 2008;322(5903):909–12. doi: 10.1126/science.1158822 ; 18988844 10.1126/science.1158822 PMC3744585

[25] Dudek M, Meng QJ. Running on time: the role of circadian clocks in the musculoskeletal system. Biochem J. 2014;463(1):1–8. doi: 10.1042/BJ20140700 ; 25195734 10.1042/BJ20140700 PMC4157581

[26] Refinetti R, Lissen GC, Halberg F. Procedures for numerical analysis of circadian rhythms. Biol Rhythm Res. 2007;38(4):275–325. doi: 10.1080/09291010600903692 ; 23710111 10.1080/09291010600903692 PMC3663600

[27] Yamazaki S, Numano R, Abe M, Hida A, Takahashi R, Ueda M, et al Resetting central and peripheral circadian oscillators in transgenic rats. Science. 2000;288(5466):682–5. . 10784453 10.1126/science.288.5466.682

[28] Garcia JA, Zhang D, Estill SJ, Michnoff C, Rutter J, Reick M, et al Impaired cued and contextual memory in NPAS2-deficient mice. Science. 2000;288(5474):2226–30. . 10864874 10.1126/science.288.5474.2226

[29] Zhang B, Horvath S. A general framework for weighted gene co-expression network analysis. Stat Appl Genet Mol Biol. 2005;4:Article17 doi: 10.2202/1544-6115.1128 . 16646834 10.2202/1544-6115.1128

[30] Langfelder P, Zhang B, Horvath S. Defining clusters from a hierarchical cluster tree: the Dynamic Tree Cut package for R. Bioinformatics. 2008;24(5):719–20. doi: 10.1093/bioinformatics/btm563 . 18024473 10.1093/bioinformatics/btm563

[31] Dong J, Horvath S. Understanding network concepts in modules. BMC Syst Biol. 2007;1:24 doi: 10.1186/1752-0509-1-24 ; 17547772 10.1186/1752-0509-1-24 PMC3238286

[32] Horvath S, Dong J. Geometric interpretation of gene coexpression network analysis. PLoS Comput Biol. 2008;4(8):e1000117 doi: 10.1371/journal.pcbi.1000117 ; 18704157 10.1371/journal.pcbi.1000117 PMC2446438

[33] Aita H, Att W, Ueno T, Yamada M, Hori N, Iwasa F, et al Ultraviolet light-mediated photofunctionalization of titanium to promote human mesenchymal stem cell migration, attachment, proliferation and differentiation. Acta Biomater. 2009;5(8):3247–57. doi: 10.1016/j.actbio.2009.04.022 . 19427421 10.1016/j.actbio.2009.04.022

[34] Li G, Cao H, Zhang W, Ding X, Yang G, Qiao Y, et al Enhanced Osseointegration of Hierarchical Micro/Nanotopographic Titanium Fabricated by Microarc Oxidation and Electrochemical Treatment. ACS Appl Mater Interfaces. 2016;8(6):3840–52. doi: 10.1021/acsami.5b10633 . 26789077 10.1021/acsami.5b10633

[35] Marycz K, Krzak J, Maredziak M, Tomaszewski KA, Szczurek A, Moszak K. The influence of metal-based biomaterials functionalized with sphingosine-1-phosphate on the cellular response and osteogenic differentaion potenial of human adipose derived mesenchymal stem cells in vitro. J Biomater Appl. 2016;30(10):1517–33. doi: 10.1177/0885328216628711 . 26801473 10.1177/0885328216628711

[36] Yamazaki S, Yoshikawa T, Biscoe EW, Numano R, Gallaspy LM, Soulsby S, et al Ontogeny of circadian organization in the rat. J Biol Rhythms. 2009;24(1):55–63. doi: 10.1177/0748730408328438 ; 19150929 10.1177/0748730408328438 PMC2665126

[37] Thalji GN, Nares S, Cooper LF. Early molecular assessment of osseointegration in humans. Clin Oral Implants Res. 2014;25(11):1273–85. doi: 10.1111/clr.12266 . 24118318 10.1111/clr.12266

[38] Nishimura I. Genetic networks in osseointegration. J Dent Res. 2013;92(12 Suppl):109S–18S. doi: 10.1177/0022034513504928 ; 24158334 10.1177/0022034513504928 PMC3827622

[39] Honda Y, Ding X, Mussano F, Wiberg A, Ho CM, Nishimura I. Guiding the osteogenic fate of mouse and human mesenchymal stem cells through feedback system control. Sci Rep. 2013;5(3):3420. 10.1038/srep03420 PMC3851880 24305548

[40] Dennis G Jr., Sherman BT, Hosack DA, Yang J, Gao W, Lane HC, et al DAVID: Database for Annotation, Visualization, and Integrated Discovery. Genome Biol. 2003;4(5):P3 . 12734009 PMC193660

[41] Qin Y, Wang L, Gao Z, Chen G, Zhang C. Bone marrow stromal/stem cell-derived extracellular vesicles regulate osteoblast activity and differentiation in vitro and promote bone regeneration in vivo. Sci Rep. 2016;25(6):21961. 10.1038/srep21961 PMC4766421 26911789

[42] Chen YG, Mantalaris A, Bourne P, Keng P, Wu JH. Expression of mPer1 and mPer2, two mammalian clock genes, in murine bone marrow. Biochem Biophys Res Commun. 2000;276(2):724–8. doi: 10.1006/bbrc.2000.3536 . 11027538 10.1006/bbrc.2000.3536

[43] Zvonic S, Ptitsyn AA, Kilroy G, Wu X, Conrad SA, Scott LK, et al Circadian oscillation of gene expression in murine calvarial bone. J Bone Miner Res. 2007;22(3):357–65. doi: 10.1359/jbmr.061114 . 17144790 10.1359/jbmr.061114

[44] Zhang Y, Hassan MQ, Li ZY, Stein JL, Lian JB, van Wijnen AJ, et al Intricate gene regulatory networks of helix-loop-helix (HLH) proteins support regulation of bone-tissue related genes during osteoblast differentiation. J Cell Biochem. 2008;105(2):487–96. doi: 10.1002/jcb.21844 ; 18655182 10.1002/jcb.21844 PMC2612593

[45] McElderry JD, Zhu P, Mroue KH, Xu J, Pavan B, Fang M, et al Crystallinity and compositional changes in carbonated apatites: Evidence from P solid-state NMR, Raman, and AFM analysis. J Solid State Chem. 2013;206 doi: 10.1016/j.jssc.2013.08.011 ; 24273344 10.1016/j.jssc.2013.08.011 PMC3835554

[46] Fretwurst T, Grunert S, Woelber JP, Nelson K, Semper-Hogg W. Vitamin D deficiency in early implant failure: two case reports. Int J Implant Dent. 2016;2(1):24 doi: 10.1186/s40729-016-0056-0 ; 27888492 10.1186/s40729-016-0056-0 PMC5124022

[47] Bryce G, MacBeth N. Vitamin D deficiency as a suspected causative factor in the failure of an immediately placed dental implant: a case report. J R Nav Med Serv. 2014;100(3):328–32. . 25895415

[48] Cermakian N, Sassone-Corsi P. Multilevel regulation of the circadian clock. Nat Rev Mol Cell Biol. 2000;1(1):59–67. doi: 10.1038/35036078 . 11413490 10.1038/35036078

[49] Zhang EE, Kay SA. Clocks not winding down: unravelling circadian networks. Nat Rev Mol Cell Biol. 2010;11(11):764–76. doi: 10.1038/nrm2995 . 20966970 10.1038/nrm2995

[50] Yang X, Downes M, Yu RT, Bookout AL, He W, Straume M, et al Nuclear receptor expression links the circadian clock to metabolism. Cell. 2006;126(4):801–10. doi: 10.1016/j.cell.2006.06.050 . 16923398 10.1016/j.cell.2006.06.050

[51] Gutierrez-Monreal MA, Cuevas-Diaz Duran R, Moreno-Cuevas JE, Scott SP. A role for 1alpha,25-dihydroxyvitamin d3 in the expression of circadian genes. J Biol Rhythms. 2014;29(5):384–8. doi: 10.1177/0748730414549239 . 25231949 10.1177/0748730414549239

[52] Seuter S, Pehkonen P, Heikkinen S, Carlberg C. The gene for the transcription factor BHLHE40/DEC1/stra13 is a dynamically regulated primary target of the vitamin D receptor. J Steroid Biochem Mol Biol. 2013;136:62–7. doi: 10.1016/j.jsbmb.2012.11.011 . 23220548 10.1016/j.jsbmb.2012.11.011

[53] Fu L, Patel MS, Bradley A, Wagner EF, Karsenty G. The molecular clock mediates leptin-regulated bone formation. Cell. 2005;122(5):803–15. doi: 10.1016/j.cell.2005.06.028 . 16143109 10.1016/j.cell.2005.06.028

[54] Bertolucci C, Cavallari N, Colognesi I, Aguzzi J, Chen Z, Caruso P, et al Evidence for an overlapping role of CLOCK and NPAS2 transcription factors in liver circadian oscillators. Mol Cell Biol. 2008;28(9):3070–5. doi: 10.1128/MCB.01931-07 ; 18316400 10.1128/MCB.01931-07 PMC2293078

[55] Hinoi E, Ueshima T, Hojo H, Iemata M, Takarada T, Yoneda Y. Up-regulation of per mRNA expression by parathyroid hormone through a protein kinase A-CREB-dependent mechanism in chondrocytes. J Biol Chem. 2006;281(33):23632–42. doi: 10.1074/jbc.M512362200 . 16777848 10.1074/jbc.M512362200

[56] Aton SJ, Colwell CS, Harmar AJ, Waschek J, Herzog ED. Vasoactive intestinal polypeptide mediates circadian rhythmicity and synchrony in mammalian clock neurons. Nat Neurosci. 2005;8(4):476–83. doi: 10.1038/nn1419 ; 15750589 10.1038/nn1419 PMC1628303

[57] Bass J. Circadian topology of metabolism. Nature. 2012;491(7424):348–56. doi: 10.1038/nature11704 . 23151577 10.1038/nature11704

[58] Yamamoto T, Nakahata Y, Tanaka M, Yoshida M, Soma H, Shinohara K, et al Acute physical stress elevates mouse period1 mRNA expression in mouse peripheral tissues via a glucocorticoid-responsive element. J Biol Chem. 2005;280(51):42036–43. doi: 10.1074/jbc.M509600200 . 16249183 10.1074/jbc.M509600200

[59] Koyanagi S, Okazawa S, Kuramoto Y, Ushijima K, Shimeno H, Soeda S, et al Chronic treatment with prednisolone represses the circadian oscillation of clock gene expression in mouse peripheral tissues. Mol Endocrinol. 2006;20(3):573–83. doi: 10.1210/me.2005-0165 . 16269518 10.1210/me.2005-0165

[60] Park EJ, Song YH, Hwang MJ, Song HJ, Park YJ. Surface Characterization and Osteoconductivity Evaluation of Micro/Nano Surface Formed on Titanium Using Anodic Oxidation Combined with H2O2 Etching and Hydrothermal Treatment. J Nanosci Nanotechnol. 2015;15(8):6133–6. . 26369213 10.1166/jnn.2015.10469

[61] Roest R, Latella BA, Heness G, Ben-Nissan B. Adhesion of sol-gel derived hydroxyapatite nanocoatings on anodised pure titanium and titanium (Ti6Al4V) alloy substrates. Surf Coat Tech. 2011;205(11):3520–9. doi: 10.1016/j.surfcoat.2010.12.030

[62] Wang G, Li J, Lv K, Zhang W, Ding X, Yang G, et al Surface thermal oxidation on titanium implants to enhance osteogenic activity and in vivo osseointegration. Sci Rep. 2016;6:31769 doi: 10.1038/srep31769 ; 27546196 10.1038/srep31769 PMC4992888

[63] Topoglidis E, Discher BM, Moser CC, Dutton PL, Durrant JR. Functionalizing nanocrystalline metal oxide electrodes with robust synthetic redox proteins. Chembiochem. 2003;4(12):1332–9. doi: 10.1002/cbic.200300707 . 14661276 10.1002/cbic.200300707

[64] Stromberg JR, Wnuk JD, Pinlac RA, Meyer GJ. Multielectron transfer at heme-functionalized nanocrystalline TiO2: reductive dechlorination of DDT and CCl4 forms stable carbene compounds. Nano Lett. 2006;6(6):1284–6. doi: 10.1021/nl060646a . 16771595 10.1021/nl060646a

[65] Dioum EM, Rutter J, Tuckerman JR, Gonzalez G, Gilles-Gonzalez MA, McKnight SL. NPAS2: a gas-responsive transcription factor. Science. 2002;298(5602):2385–7. doi: 10.1126/science.1078456 . 12446832 10.1126/science.1078456

[66] Ishida M, Ueha T, Sagami I. Effects of mutations in the heme domain on the transcriptional activity and DNA-binding activity of NPAS2. Biochem Biophys Res Commun. 2008;368(2):292–7. doi: 10.1016/j.bbrc.2008.01.053 . 18230344 10.1016/j.bbrc.2008.01.053

