# Supplementary material for: Titanium biomaterials with complex surfaces induced aberrant peripheral circadian rhythms in bone marrow mesenchymal stromal cells
Source: PLoS One. 2017 Aug 17;12(8):e0183359. doi: 10.1371/journal.pone.0183359 (PMC5560683; doi:10.1371/journal.pone.0183359)

Hassan et al. Titanium biomaterials with complex surfaces induced aberrant peripheral circadian rhythms in bone marrow mesenchymal stromal cells

S1 Fig. Surface characterization of Ti substrates by SEM. **A**. Commercially pure Ti discs were produced by a rotary machine, which left the isotropic grooves. **B**. The machined surface was treated with double acid etching (DAE), which generated submicron levels of anisotropic rough surface. **C**. The discrete crystalline deposition (DCD) surface modification to the DAE surface created the less than 50% surface coverage of hydroxyapatite nanoparticles (2~10 nanometer). **D**. To eliminate the effect of surface topography and to evaluate the role of Ti chemistry, the machined Ti disc was polished until 600-grade. **E**. Prior to DAE-DCD surface treatment, Ti disc was treated with sand blasting (B), which gave rise to greater surface roughness at the submicron to micron levels. **F**. The B-DAE-DCD surface modification remained to contain hydroxyapatite nanoparticles up to 50% surface area. The machined surface Ti implant is used as dental implant. Clinically used dental implants are also available with DAE, DAE-DCD and B-DAE-DCD surfaces (Zimmer Biomet, Palm Beach Garden, FL).

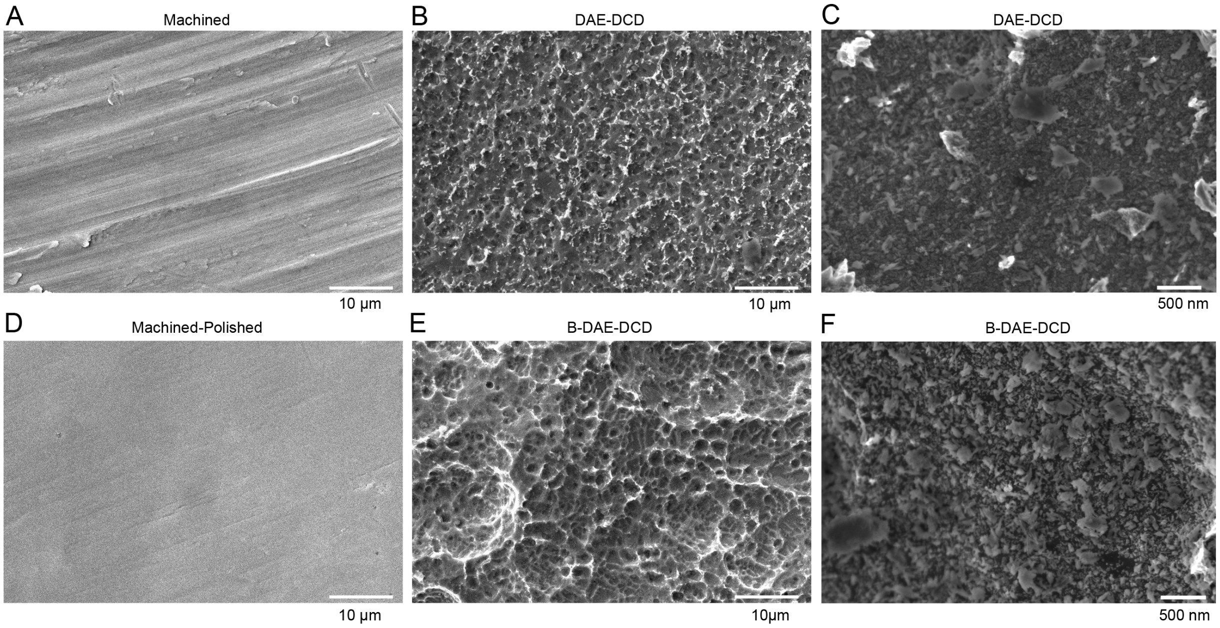

Supplement: S1 Fig — (PDF) [file pone.0183359.s001.pdf]
