# Supplementary material for: Titanium biomaterials with complex surfaces induced aberrant peripheral circadian rhythms in bone marrow mesenchymal stromal cells
Source: PLoS One. 2017 Aug 17;12(8):e0183359. doi: 10.1371/journal.pone.0183359 (PMC5560683; doi:10.1371/journal.pone.0183359)

Hassan et al. Titanium biomaterials with complex surfaces induced aberrant peripheral circadian rhythms in bone marrow mesenchymal stromal cells

S2 Fig. Ti discs fit in 35 mm culture dish used for this study. Bone marrow stromal cells (BMSC) were cultured on the Ti discs. In luminometer experiments, 35 mm dish was sealed. Rat femur BMSC cultured on the test substrate surfaces with osteogenic medium demonstrated *in vitro* mineralization. *: $p<0.05$

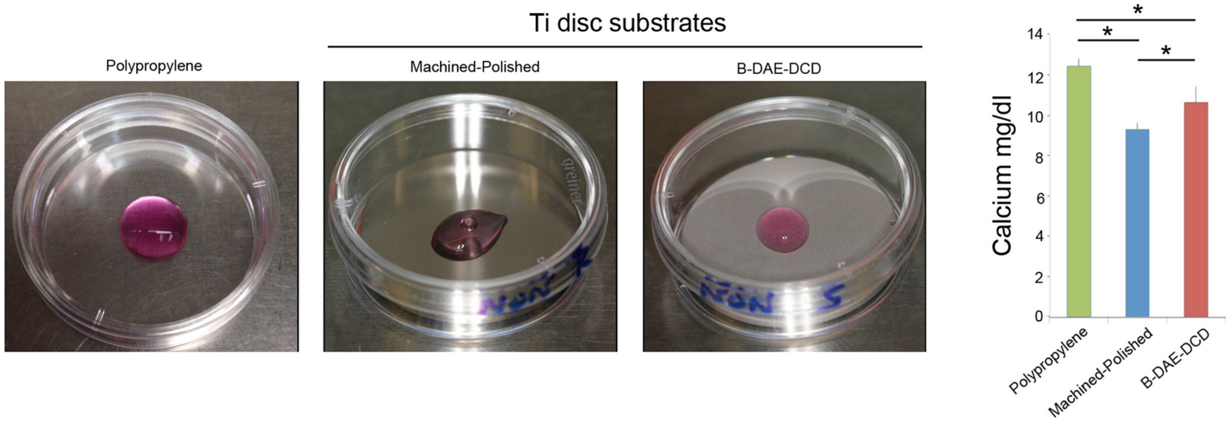

Supplement: S2 Fig — (PDF) [file pone.0183359.s002.pdf]
