# Supplementary material for: Titanium biomaterials with complex surfaces induced aberrant peripheral circadian rhythms in bone marrow mesenchymal stromal cells
Source: PLoS One. 2017 Aug 17;12(8):e0183359. doi: 10.1371/journal.pone.0183359 (PMC5560683; doi:10.1371/journal.pone.0183359)

Hassan et al. Titanium biomaterials with complex surfaces induced aberrant peripheral circadian rhythms in bone marrow mesenchymal stromal cells

S3 Fig. The cell viability test of BMSC in the luminometer culture condition. The moderate decrease of cell viability was unlikely to be the major cause of *Per1::luc* expression modulation in the B-DAE-DCD group.

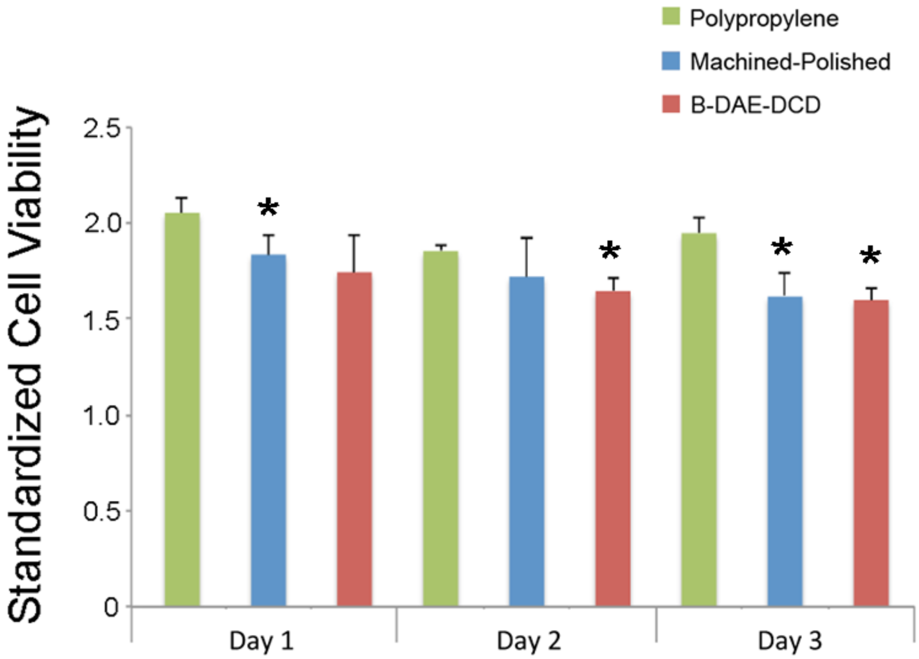

Supplement: S3 Fig — (PDF) [file pone.0183359.s003.pdf]
