# Supplementary material for: Titanium biomaterials with complex surfaces induced aberrant peripheral circadian rhythms in bone marrow mesenchymal stromal cells
Source: PLoS One. 2017 Aug 17;12(8):e0183359. doi: 10.1371/journal.pone.0183359 (PMC5560683; doi:10.1371/journal.pone.0183359)

Hassan et al. Titanium biomaterials with complex surfaces induced aberrant peripheral circadian rhythms in bone marrow mesenchymal stromal cells

S4 Fig. Circadian rhythm-related gene expression by BMSC cultured on Ti discs with machined or B-DAE-DCD surface, as well as Zirconia disc. The gene expression pattern was similar in those on machined Ti disc and Zirconia disc.

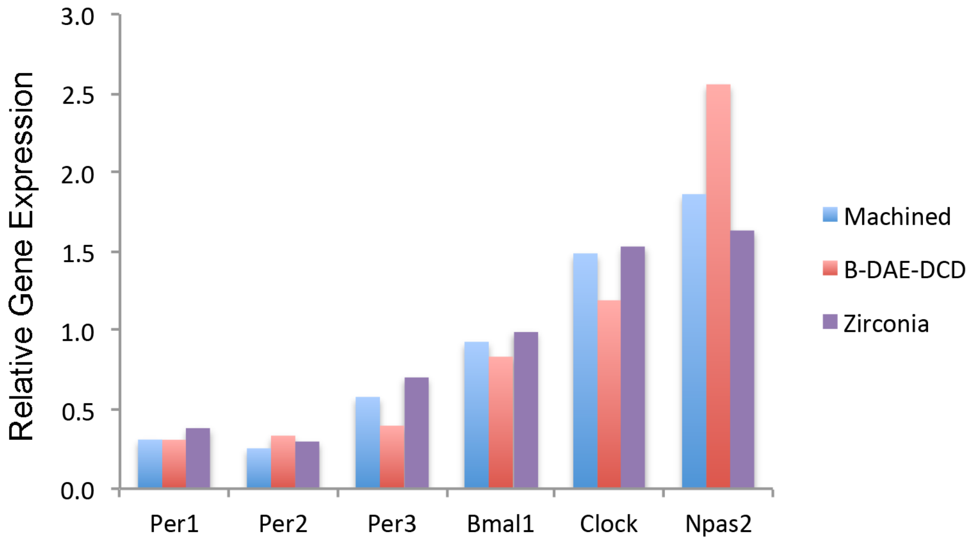

Supplement: S4 Fig — (PDF) [file pone.0183359.s004.pdf]
