# Supplementary material for: Titanium biomaterials with complex surfaces induced aberrant peripheral circadian rhythms in bone marrow mesenchymal stromal cells
Source: PLoS One. 2017 Aug 17;12(8):e0183359. doi: 10.1371/journal.pone.0183359 (PMC5560683; doi:10.1371/journal.pone.0183359)
Supplement: S1 Table — (PDF) [file pone.0183359.s005.pdf]

Hassan et al. Titanium biomaterials with complex surfaces induced aberrant peripheral circadian rhythms in bone marrow mesenchymal stromal cells

S1 Table Cosinor-based rhythmometry analysis of circadian rhythm gene expression in human BMSC cultured on polypropylene dish, machined Ti disc or B-DAE-DCD disc (24h fixed cosine curve)

Per1

	Polypropylene	Machined	B-DAE-DCD
**Amplitude**	0.3206	0.1629	0.2288
**Acrophase (degree)**	178	180	225
**Acrophase (hour)**	15.84	16	19.01

Per2

	Polypropylene	Machined	B-DAE-DCD
**Amplitude**	0.5684	0.4021	0.8095
**Acrophase (degree)**	217	233	258
**Acrophase (hour)**	18.45	19.52	21.19

Per3

	Polypropylene	Machined	B-DAE-DCD
**Amplitude**	0.5539	0.3104	0.6788
**Acrophase (degree)**	207	208	249
**Acrophase (hour)**	17.79	17.84	20.58
