# Supplementary material for: Titanium biomaterials with complex surfaces induced aberrant peripheral circadian rhythms in bone marrow mesenchymal stromal cells
Source: PLoS One. 2017 Aug 17;12(8):e0183359. doi: 10.1371/journal.pone.0183359 (PMC5560683; doi:10.1371/journal.pone.0183359)
Supplement: S2 Table — (PDF) [file pone.0183359.s006.pdf]

Hassan et al. Titanium biomaterials with complex surfaces induced aberrant peripheral circadian rhythms in bone marrow mesenchymal stromal cells

S2 Table Hub genes in Blue module

Gene ID	Gene Name	Number of Connections	Protein Function
A_44_P214873	Arntl	23	Circadian rhythm, E-box binding
A_44_P364620, A_43_P12768	Bhlhe40	41, 42	
A_43_P15357	Bhlhe41	36	
A_43_P15473	Clock	22	
A_42_P515454, A_44_P192988	Csnk1e	26, 26	
A_44_P405793	Npas2	32	
A_44_P264477	Pacsin1	34	
A_44_P175041	Nr1d1	30	Nuclear steroid hormone receptors, DNA binding
A_44_P395572	Nr1d2	29	
A_43_P14310	Nr2f1	24	
A_44_P421333	Nr2f2	25	
A_44_P243231	Nr2f6	42	
A_44_P522524	Nr4a2	44	
A_44_P654444, A_43_P12619, A_43_P11932	Nr4a3	43, 38, 31	
A_44_P250983, A_44_P191287	Nrbp2	42, 42	
A_42_P812008	P8	37	
A_44_P468153	Thrb	28	
A_44_P276492	Vdr	35	Vitamin D related
A_44_P118625	Calb1	41	
A_43_P15847	Cyp27a1	31	
A_43_P12154	Acan	25	Cartilage-related extracellular matrix
A_43_P11684	Alpl	43	
A_44_P295789	Comp	28	
A_42_P636281	Dmp1	35	
A_44_P241156, A_44_P357420	Mamdc2	42, 43	
A_44_P322497	Serpinh1	44	
A_44_P515798	Ucma	13	
A_43_P10333	Efemp1	25	Others
A_42_P669820	Efemp2	43	
A_42_P623839, A_44_P271347	Frem1	41, 42	
A_42_P624773	Cpz	32	
A_42_P527961	Pmch	30	
A_43_P13420, A_44_P233867	Spon1	21, 26	
A_44_P421417	Spon2	35	
A_44_P321176	Hr	26	
A_44_P1029131	Il33	22	
