# Supplementary material for: Titanium biomaterials with complex surfaces induced aberrant peripheral circadian rhythms in bone marrow mesenchymal stromal cells
Source: PLoS One. 2017 Aug 17;12(8):e0183359. doi: 10.1371/journal.pone.0183359 (PMC5560683; doi:10.1371/journal.pone.0183359)
Supplement: S3 Table — (PDF) [file pone.0183359.s007.pdf]

Hassan et al. Titanium biomaterials with complex surfaces induced aberrant peripheral circadian rhythms in bone marrow mesenchymal stromal cells

S3 Table Kyoto Encyclopedia of Genes and Genomes (KEGG) analysis of Blue module hub genes

#pathway ID	pathway description	observed gene count	false discovery rate	matching proteins in your network (labels)
4710	Circadian rhythm	7	5.10E-12	Arntl,Bhlhe40,Bhlhe41,Clock,Csnk1e,Npas2,Nr1d1
