# Supplementary material for: Titanium biomaterials with complex surfaces induced aberrant peripheral circadian rhythms in bone marrow mesenchymal stromal cells
Source: PLoS One. 2017 Aug 17;12(8):e0183359. doi: 10.1371/journal.pone.0183359 (PMC5560683; doi:10.1371/journal.pone.0183359)
Supplement: S4 Table — (PDF) [file pone.0183359.s008.pdf]

Hassan et al. Titanium biomaterials with complex surfaces induced aberrant peripheral circadian rhythms in bone marrow mesenchymal stromal cells

S4 Table: Gene ontogeny analysis (Function) of Blue module hub genes

#pathway ID	pathway description	observed gene count	false discovery rate	matching proteins in your network (labels)
GO.0003707	steroid hormone receptor activity	8	3.21E-12	Nr1d1,Nr1d2,Nr2f2,Nr2f6,Nr4a2,Nr4a3,Thrb,Vdr
GO.0004879	RNA polymerase II transcription factor activity, ligand-activated sequence-specific DNA binding	6	2.88E-09	Nr2f2,Nr2f6,Nr4a2,Nr4a3,Thrb,Vdr
GO.0043565	sequence-specific DNA binding	10	9.15E-09	Arntl,Clock,Nr1d1,Nr1d2,Nr2f2,Nr2f6,Nr4a2,Nr4a3,Thrb,Vdr
GO.0003700	transcription factor activity, sequence-specific DNA binding	9	1.27E-07	Arntl,Clock,Nr1d1,Nr1d2,Nr2f2,Nr4a2,Nr4a3,Thrb,Vdr
GO.0046872	metal ion binding	16	2.89E-07	Acan,Alpl,Comp,Cpz,Cyp27a1,Efemp1,Hr,Nr1d1,Nr1d2,Nr2f2,Nr2f6,Nr4a2,Nr4a3,Spon2,Thrb,Vdr
GO.0008270	zinc ion binding	10	3.24E-07	Calb1,Cpz,Nr1d1,Nr1d2,Nr2f2,Nr2f6,Nr4a2,Nr4a3,Thrb,Vdr
GO.0046914	transition metal ion binding	11	3.93E-07	Calb1,Cpz,Cyp27a1,Nr1d1,Nr1d2,Nr2f2,Nr2f6,Nr4a2,Nr4a3,Thrb,Vdr
GO.0003677	DNA binding	10	6.03E-06	Arntl,Bhlhe40,Clock,Hr,Nr1d1,Nr1d2,Nr2f2,Nr2f6,Nr4a2,Vdr
GO.0005499	vitamin D binding	3	1.07E-05	Calb1,Comp,Vdr
GO.0043167	ion binding	17	2.06E-05	Acan,Alpl,Comp,Cpz,Cxcl10,Cyp27a1,Efemp1,Hr,Nr1d1,Nr1d2,Nr2f6,Nr4a2,Nr4a3,Pacsin1,Spon2,Thrb,Vdr

GO.0044212	transcription regulatory region DNA binding	6	7.97E-05	Arntl,Bhlhe40,Clock,Nr1d1,Nr1d2,Nr4a2
GO.0004871	signal transducer activity	9	0.000111	Arntl,Clock,Efemp1,Nr1d1,Nr1d2,Nr2f2,Nr2f6,Nr4a2,Nr4a3
GO.0001047	core promoter binding	4	0.000156	Arntl,Clock,Nr1d1,Nr1d2
GO.0070888	E-box binding	3	0.000493	Arntl,Bhlhe40,Clock
GO.0097159	organic cyclic compound binding	13	0.00173	Arntl,Bhlhe40,Calb1,Clock,Comp,Cyp27a1,Hr,Nr1d1,Nr1d2,Nr2f2,Nr2f6,Nr4a2,Vdr
GO.0038023	signaling receptor activity	7	0.00229	Efemp1,Nr1d1,Nr1d2,Nr2f2,Nr2f6,Nr4a2,Nr4a3
GO.0000976	transcription regulatory region sequence-specific DNA binding	4	0.00829	Arntl,Nr1d1,Nr1d2,Nr4a2
GO.0005102	receptor binding	6	0.014	Cxcl10,Efemp1,Hr,Il33,Pmch,Spon2
GO.0005515	protein binding	11	0.0181	Comp,Cxcl10,Efemp1,Hr,Il33,Nr1d1,Pacsin1,Pmch,Serpinh1,Spon2,Thrb
GO.0008289	lipid binding	5	0.0181	Calb1,Comp,Nr2f2,Pacsin1,Vdr
GO.1901363	heterocyclic compound binding	11	0.0266	Arntl,Bhlhe40,Clock,Cyp27a1,Hr,Nr1d1,Nr1d2,Nr2f2,Nr2f6,Nr4a2,Vdr
GO.0005539	glycosaminoglycan binding	3	0.046	Acan,Comp,Cxcl10
