# Supplementary material for: Titanium biomaterials with complex surfaces induced aberrant peripheral circadian rhythms in bone marrow mesenchymal stromal cells
Source: PLoS One. 2017 Aug 17;12(8):e0183359. doi: 10.1371/journal.pone.0183359 (PMC5560683; doi:10.1371/journal.pone.0183359)
Supplement: S5 Table — (PDF) [file pone.0183359.s009.pdf]

Hassan et al. Titanium biomaterials with complex surfaces induced aberrant peripheral circadian rhythms in bone marrow mesenchymal stromal cells

S5 Table ITV+ vs. OSV+ trait comparison. *: p<0.05

Modlule	Trait	p value
Blue	0.96	0.0004*
Yellow 4	0.78	0.02*
Light green	0.64	0.09
Bisque 4	0.6	0.1
Dark seagreen 4	0.58	0.1
Sienna 3	0.58	0.1
Black	0.57	0.1
Green	0.56	0.1
Pink	0.55	0.2
Dark orange 2	0.51	0.2
Light cyan	0.47	0.2
Thistle 2	0.46	0.3
Dark grey	0.39	0.3
Antique white 4	0.38	0.4
Thistle 1	0.33	0.4
Royal blue	0.3	0.5
Light yellow	0.29	0.5
Honeydew 1	0.29	0.5
Navaho white 2	0.27	0.5
Maroon	0.25	0.6
Red	0.24	0.6
Dark magenta	0.23	0.6
Skyblue	0.23	0.6
Floral white	0.22	0.6
Medium orchid	0.21	0.6
Dark red	0.21	0.6
Skyblue 2	0.21	0.6
Light cyan 1	0.2	0.6
Dark turquoise	0.2	0.6
Violet	0.11	0.8
Dark slateblue	-0.015	1
Coral 1	-0.07	0.9
Salmon	-0.077	0.9
Salmon 4	-0.08	0.9
Lavender blush 3	-0.084	0.8
Orange	-0.089	0.8
Ivory	-0.091	0.8
Plum 1	-0.11	0.8
Pale turquoise	-0.17	0.7

Hassan et al. Titanium biomaterials with complex surfaces induced aberrant peripheral circadian rhythms in bone marrow mesenchymal stromal cells

Orange red 4	-0.2	0.6
Grey	-0.22	0.6
Midnight blue	-0.23	0.6
Brown 4	-0.23	0.6
Brown	-0.23	0.6
Pale vilet red 3	-0.25	0.6
Medium purple 3	-0.25	0.6
Purple	-0.28	0.5
Yellow green	-0.32	0.4
Dark green	-0.33	0.4
White	-0.42	0.3
Light Steel blue 1	-0.43	0.3
Plum 2	-0.43	0.3
Tan	-0.43	0.3
Magenta	-0.44	0.3
Dark orange	-0.45	0.3
Light pink 4	-0.46	0.2
Dark olive green	-0.46	0.2
Steelblue	-0.46	0.2
Cyan	-0.47	0.2
Coral 2	-0.51	0.2
Green yellow	-0.52	0.2
Yellow	-0.55	0.2
Saddle brown	-0.61	0.1
Skyblue 3	-0.65	0.08
Grey 60	-0.8	0.02*
turquoise	-0.94	0.000004*
