# Supplementary material for: Titanium biomaterials with complex surfaces induced aberrant peripheral circadian rhythms in bone marrow mesenchymal stromal cells
Source: PLoS One. 2017 Aug 17;12(8):e0183359. doi: 10.1371/journal.pone.0183359 (PMC5560683; doi:10.1371/journal.pone.0183359)
Supplement: S6 Table — (PDF) [file pone.0183359.s010.pdf]

Hassan et al. Titanium biomaterials with complex surfaces induced aberrant peripheral circadian rhythms in bone marrow mesenchymal stromal cells

S6 Table ITV+ vs. ITV- trait comparison. *: $p<0.05$

Module	Trait	p value
Dark turquoise	0.83	0.01*
Blue	0.63	0.09
Green	0.59	0.1
Green yellow	0.58	0.1
Pink	0.56	0.1
Magenta	0.55	0.2
Light cyan	0.55	0.2
Salmon	0.53	0.2
Tan	0.52	0.2
Pale turquoise	0.51	0.2
Plum 1	0.44	0.3
Dark orange	0.41	0.3
Navajo white 2	0.38	0.4
Saddle brown	0.36	0.4
Maroon	0.34	0.4
Skyblue 2	0.34	0.4
Antique white 4	0.32	0.4
Bisque 4	0.29	0.5
Light green	0.26	0.5
Medium orchid	0.25	0.6
Plum 2	0.22	0.6
Brown 4	0.2	0.6
Dark slate blue	0.2	0.6
Skyblue	0.17	0.7
Brown	0.16	0.7
Light yellow	0.14	0.7
Medium purple 2	0.14	0.7
Light steele blue	0.12	0.8
Medium purple 3	0.11	0.8
Plum	0.089	0.8
Dark olive green	0.062	0.9
Grey 60	0.056	0.9
Orange red 4	0.053	0.9
Coral 2	0.051	0.9
Light cyan 1	-0.0014	1
Dark sea green 4	-0.026	1
Yellow	-0.063	0.9
Lavender blush 3	-0.074	0.9
Coral 1	-0.086	1

Hassan et al. Titanium biomaterials with complex surfaces induced aberrant peripheral circadian rhythms in bone marrow mesenchymal stromal cells

Violet	-0.1	0.8
Dark orange 2	-0.12	0.8
Dark grey	-0.12	0.8
Dark magenta	-0.14	0.7
Skyblue 1	-0.15	0.7
Dark green	-0.16	0.7
Orange	-0.19	0.6
Grey	-0.22	0.6
Sky blue 3	-0.23	0.6
Light pink 4	-0.23	0.6
Black	-0.24	0.6
Midnight blue	-0.26	0.5
Thistle 1	-0.35	0.4
Orange red 3	-0.35	0.4
Steel blue	-0.37	0.4
Floral white	-0.38	0.4
Light steel blue 1	-0.39	0.3
Dark red	-0.42	0.3
Sienna 3	-0.43	0.3
Royal blue	-0.47	0.2
Yellow 4	-0.48	0.2
Yellow green	-0.48	0.2
Thistle 2	-0.49	0.2
Red	-0.5	0.2
Hoenydew 1	-0.5	0.2
Salmon 4	-0.55	0.2
Turquoise	-0.6	0.1
Pale violet red 3	-0.65	0.08
White	-0.66	0.07
Cyan	-0.75	0.03*
Ivory	-0.83	0.01*
Purple	-0.95	0.0004*
